# RiboScreen^TM^ Technology Delivers Small-Molecule Ribodrugs to Convert Ribosomal Proteins into Molecular Valves for Tailored Protein Production Levels in Rare and Prevalent Disease †

**DOI:** 10.3390/biomedicines14071419

**Published:** 2026-06-23

**Authors:** Genevieve Edobor, Ronald Huber, Christoph Reiter, Hanna Gercke, Niklas Kaefer, Elli Kronsteiner, Bjoern Wimmer, Marlies Wimmer, Thomas Karl, Mark Rinnerthaler, Jan Krauß, Heinrich Krobath, Thomas Mohr, Christopher Gerner, Joerg von Hagen, Norbert Müller, Helmut Hintner, Bernadette Liemberger, Ulrich Koller, Johann W. Bauer, Gazmend Temaj, Hannelore Breitenbach-Koller

**Affiliations:** 1 Department of Biosciences and Medical Biology, University of Salzburg, 5020 Salzburg, Austria; edoborgene@gmail.com (G.E.); ronald.huber@stud.plus.ac.at (R.H.); christoph.reiter@plus.ac.at (C.R.); hanna.gercke@stud.plus.ac.at (H.G.); niklas.kaefer@plus.ac.at (N.K.); elli.kronsteiner@stud.plus.ac.at (E.K.); publishingbw@outlook.com (B.W.); marlies.wimmer@outlook.de (M.W.); thomas.karl@plus.ac.at (T.K.); mark.rinnerthaler@plus.ac.at (M.R.); j.krauss@kbhb.at (J.K.); 2 KBHB GmbH, Franz-Rehrl Platz 2, 5020 Salzburg, Austria; 3 Institute for Theoretical Physics, Johannes Kepler University Linz, 4020 Linz, Austria; heinrich.krobath@jku.at; 4 Department of Analytical Chemistry, Faculty of Chemistry, University of Vienna, 1090 Vienna, Austria; thomas.mohr@univie.ac.at (T.M.); christopher.gerner@univie.ac.at (C.G.); 5 Join Metabolome Facility, University of Vienna, Waehringer Straße. 38, 1090 Vienna, Austria; 6 Merck KGaA Healthcare, Frankfurter Straße 250, 64293 Darmstadt, Germany; joerg.von.hagen@merckgroup.com; 7 ryon-Greentech Accelerator, Mainzer Straße 41, 64579 Gernsheim, Germany; 8 Institute of Biochemistry, Johannes Kepler University Linz, 4020 Linz, Austria; norbert.mueller@jku.at; 9 Department of Chemistry, Faculty of Science, University of South Bohemia in Českých Budějovicích, Branišovská 1760, 370 05 České Budějovice, Czech Republic; 10 Department of Dermatology and Allergology, University Hospital Salzburg, Muellner Hauptstraße 48, 5020 Salzburg, Austria; hhintner@icloud.com (H.H.); joh.bauer@salk.at (J.W.B.); 11 EB House Austria, Research Program for Molecular Therapy of Genodermatoses, Department of Dermatology and Allergology, University Hospital of the Paracelsus Medical University Salzburg, Muellner Hauptstraße 48, 5020 Salzburg, Austria; b.liemberger@salk.at (B.L.); u.koller@salk.at (U.K.); 12 Faculty of Pharmacy, College UBT, 10000 Prishtina, Kosovo

**Keywords:** ribosomal proteins, novel drug targets, RiboScreen^TM^, customized protein production, precision intervention in rare and prevalent disease

## Abstract

Across all kingdoms of life, ribosomes are indispensable molecular machines that translate genetic information into the proteome of living cells. The fundamental catalytic centers of the ribosome, constructed primarily from ribosomal RNA (rRNA), exhibit remarkable conservation between the major domains of life. The ribosome’s A-site deciphers the mRNA’s triplet code, while the P-site synthesizes the growing protein chain and the E-site provides exit for deacylated tRNA; a distinct tunnel facilitates nascent polypeptide export. While the conservation of ribosomal proteins is less pronounced between bacteria and eukaryotes, striking homology exists from simple eukaryotes to humans. Ribosomal proteins were traditionally viewed mainly as scaffolding agents, steering rRNA folding during ribosome biogenesis and maintaining structural stability during translation. However, since the early 2000s, advances in structural and functional ribosome analysis have ushered in a more nuanced paradigm: ribosomes are no longer considered uniform machines. Instead, an array of rRNA and ribosomal protein modifications generates a spectrum of ribosome populations capable of specialized translation. RiboScreen^TM^ technology leverages this regulatory potential of individual ribosomal proteins, enabling deliberate modulation of target protein output and representing a promising tool for correcting dysregulated protein expression involved in rare and common diseases. This review will first introduce relevant aspects of ribosome biology and then showcase the tools of this new technology. Finally, we report examples for the delivery of small molecules to target ribosomal proteins for tailored restoration of protein production levels in rare and prevalent diseases.

## 1. Introduction

The RiboScreen™ platform technology represents a paradigm shift in drug discovery and translational medicine, enabling systemic yet targeted modulation of protein levels in both rare and widespread diseases [1,2,3,4]. By intervening in a targeted fashion and at the physiological level in the process of protein synthesis, this innovative approach holds the promise not only of disease amelioration but, in select cases, curative therapy. To contextualize the significance of RiboScreen™ technology, this review will first present a coherent overview of ribosome biology, synthesizing key concepts necessary to appreciate subsequent advances. The RiboScreen™ technological landscape is then explored, presenting the advantages and challenges of this technology. Finally, selective case examples are discussed where small molecules targeting ribosomal proteins provide tailored restoration or modulation of protein production levels in both rare and prevalent diseases.

To establish a conceptual framework for ribosome biology, this review covers (i) Ribosome composition and structural features: conserved core architecture and evolutionary diversification, (ii) genomic organization, transcriptional control, processing, and modification of ribosomal DNA (rDNA), (iii) genomic organization, transcriptional control, translational control and import into the nucleus of ribosomal proteins, (iv) ribosome biogenesis, (v) ribosome function in translation, and (vi) ribosome specialization. The focus will be on eukaryotic cytoplasmic ribosomes, as extensively characterized in budding yeast and human cells, with additional insights from bacterial and archaeal systems where relevant. While each of these areas has been extensively researched and merits its own comprehensive review, here, we emphasize key studies and recent reviews on yeast and human cytoplasmic ribosomes to provide a foundational perspective that supports understanding of the RiboScreen^TM^ technological toolkit.

Core knowledge of ribosome biology is also essential for understanding the precision mode of action of RiboScreen™ technology, which does not interfere with the intricate processes of ribosome biogenesis or the universally conserved rRNA catalytic centers, but instead acts on actively translating ribosomes by targeting surface-exposed globular domains of selected ribosomal proteins. Building on this foundation, it becomes clear why ribosomal proteins are particularly suitable entry points for precision ribodrugs: they sit at the nexus of conserved core translation and regulatory fine-tuning, allowing local modulation of specific translational events without collapsing global protein synthesis or disturbing the structural integrity of the ribosome.

This core knowledge of ribosome biology is particularly pertinent for appreciating the unique promise of the RiboScreen™ technological landscape, which is described next. Unlike approaches that disrupt global ribosome biogenesis or indiscriminate proteome output, RiboScreen™ enables highly precise modulation of the production level of a protein of interest (POI) through strategic targeting of selected ribosomal proteins devoted to tailoring the translational output of this POI. Ribosomal proteins, once considered static structural elements of the ribosome, have recently emerged as candidate dynamic regulators of post-transcriptional control, capable of adjusting protein synthesis in a highly selective manner [5,6,7]. Historically overlooked as drug targets, ribosomal proteins now represent a promising frontier for therapeutic intervention, because small molecules that bind to their globular domains can re-tune translation on active ribosomes with minimal impact on core ribosomal functions or global proteostasis. In this way, RiboScreen™ leverages decades of basic ribosome research to convert individual ribosomal proteins into molecular valves that can be opened or closed with small molecules to achieve context-specific, transcript-level precision in drug action.

RiboSceen^TM^ technology is then introduced as a platform technology. By systematically identifying target ribosomal proteins as transcript-selective translational switches and then discovering small-molecule ligands that bind accessible sites on these proteins, the RiboScreen platform integrates genetics, structure-based and AI-assisted screening, and multi-layered functional validation from yeast models to human cells, organoids and patient tissue. This stepwise precision-filter pipeline enables early elimination of non-specific compounds, rapid prioritization of ribodrugs with favorable efficacy and safety profiles, and accelerates repurposing opportunities, as illustrated by the artesunate program in severe junctional epidermolysis bullosa and the tropoelastin-boosting ligands for elastin-deficient indications. Consequently, RiboScreen emerges as a next-generation discovery engine that links fundamental ribosome biology to clinically actionable, transcript- and context-specific interventions, positioning ribosomal proteins as a new class of rationally druggable nodes in precision medicine.

In the broader landscape of drug development, RiboScreen™ complements and extends modalities such as gene therapy and RNA-targeting approaches, which aim to correct or bypass pathogenic variants at the DNA or RNA level but often face challenges in delivery, durability, and immunogenicity. By intervening at the level of translation on endogenous transcripts, RiboScreen™ offers an orthogonal route to normalize dysregulated protein expression without the need for permanent genome modification or exogenous gene addition, and can be combined with these modalities in rational therapeutic strategies. Systemic pharmacological therapies remain the “holy grail” of drug development: despite persistently high attrition rates, especially in complex or rare diseases, successful small-molecule or oral agents are inherently scalable, can be manufactured at an industrial scale, and are deployable worldwide through existing distribution channels without the highly individualized logistics of advanced therapy medical products (ATMPs). Positioning ribosomal proteins as druggable nodes within this framework, RiboScreen™ aspires to deliver systemic, globally accessible precision medicines that translate sophisticated ribosome biology into practical, scalable treatments for patients.

For a prototypic application of RiboScreen™ Technology, we present first drug development for rare disease severe Junctional Epidermolysis Bullosa (sJEB). sJEB is a rare, often lethal genetic disorder of the epithelium, primarily resulting from biallelic nonsense or premature termination codon (PTC) mutations in the LAMB3 gene. LAMB3 encodes the skin anchor protein Lamb3, the β3 chain of trimeric laminin 332, a critical component of hemidesmosomal skin anchoring structures. PTC mutations disrupt full-length protein synthesis, preventing the assembly of truncated Lamb3 into the laminin 332 complex, leading to serious clinical outcomes. These include extensive blistering at birth, periorificial erosions, mucosal surface loss, respiratory complications, and, ultimately, sepsis and failure to thrive, often culminating in early infant mortality [8]. Currently, there is no approved systemic therapy to replenish full-length Lamb3, and long-term systemic correction is urgently needed. RiboScreen™ technology has enabled the repurposing of Artesunate, a small molecule identified through this platform, which was used off-label in a sJEB patient and resulted in significant wound closure [9]. This case exemplifies the ability of RiboScreen™-guided interventions to restore function in previously intractable genetic disorders.

Next, we discuss RiboScreen^TM^ technology’s approach to foster tropoelastin Replenishment. There is a substantial unmet medical and aesthetic need for interventions that replenish tropoelastin, the precursor of elastin and elastic fibers. This would improve wound healing, enhance skin resilience during aging, and address elastin insufficiency in cardiovascular and pulmonary diseases [4]. While various strategies have been devised to supplement tropoelastin and elastin in tissues, no systemic treatments are currently available [10,11,12]. Utilizing RiboScreen™ technology, small molecules were identified via virtual docking studies that specifically bind to the target ribosomal protein L40e/rpL40e in both yeast and human systems. Experimental validation demonstrated that treatment of naïve yeast vehicles with these compounds resulted in up to a two-fold increase in tropoelastin production. These findings lay the groundwork for advancing these candidate molecules into human cellular models, with the ultimate goal of developing systemic therapies for diseases associated with elastin deficiency.

In closing, we provide an outlook on the application of RiboScreen^TM^ technology in the areas of various rare and common diseases caused by PTC mutations as well as the broader applications of the technology in cancer.

In summary, the RiboScreen™ platform exemplifies a transformative approach in precision medicine by harnessing the regulatory potential of ribosomal proteins for targeted therapeutic interventions, as demonstrated in both rare genetic diseases and broader clinical indications.

## 2. Ribosome Composition and Structural Features: Conserved Core Architecture and Evolutionary Diversification

**Figure 1:**
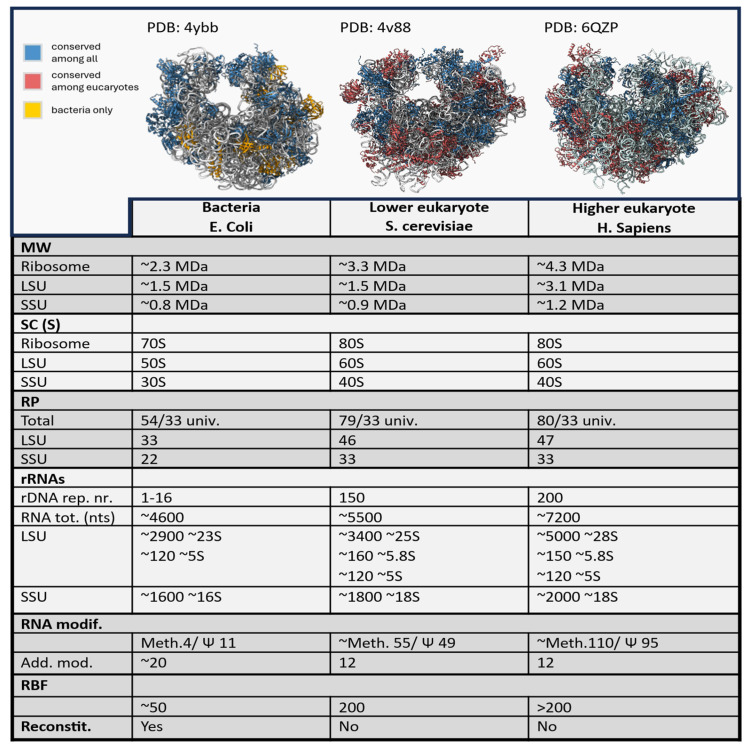
The ribosome across domains: conserved structural features and evolutionary expansion from bacteria to eukarya. Depicted are representative high-resolution structures of the bacterial 70S ribosome (*E. coli*), the yeast (*S. cerevisiae*) 80S ribosome and the human (*H. sapiens*) 80S ribosome, illustrating a conserved ribosome architecture. The accompanying table summarizes key constituents of bacterial and eukaryotic ribosomes, reporting on the expansion of ribosomal RNA and ribosomal proteins. This includes information on molecular mass (MM) sedimentation coefficients (SE) given in Svedberg units (S), numbers of ribosomal proteins (RP), rRNA species (rRNA) and total nucleotide content, rDNA repeat number (rDNA rep.nr.), as well as rRNA modifications, (RNA modif.) ribosome biogenesis factors, and reconstitution capability (Reconstit.), highlighting the compositional complexity increase from bacteria to lower and higher eukaryotes. Large ribosomal subunit (LSU), small ribosomal subunit (SSU). Ribosomal structures and data characterizing ribosomal components are reported in and modified from [19,26,27,28].

Translation-competent ribosomes, 80S ribosomes in eukaryotes and 70S ribosomes in prokaryotes, are composed of two distinct subunits, a small subunit (SSU), a 30S (bacteria) or a 40S SSU (eukaryotes), and a large subunit (LSU), a 50S (bacteria) or a 60S (eukaryotes) species, respectively (Figure 1). Note that S denotes Svedberg units, a measure for the sedimentation coefficient of a particle upon centrifugation, which is not additive upon formation of a complex from subunits. Over the past three decades, structural biology has transformed our understanding of the ribosome. Pioneering advances in X-ray crystallography and, more recently, high-resolution cryo-electron microscopy (cryo-EM) have revealed the ribosome in all domains of life as a dynamic molecular machine captured in multiple functional states. These efforts have culminated in atomic-level reconstructions across evolutionary lineages, laying the foundation for mechanistic and comparative insights that define modern ribosome research [13,14,15,16,17,18,19,20,21,22,23,24,25,26,27,28].

In eukaryotes, the 80S ribosome harbors four ribosomal RNAs (rRNAs) onto which 80 ribosomal proteins (RPs) are assembled in yeast and human ribosomes. Present-day eukaryotic ribosomes are evolutionary telescopes that reveal the genesis of ribosomes that started more than 4 billion years ago [29,30]. Ribosomal architecture in the super kingdoms of life, from bacteria to archaea to Eukarya, is built on the universal common core, a 2 MDa structure established by the Last Universal Common Ancestor (LUCA) [29]. The ribosome entered stasis after LUCA and remained there for billions of years, with bacteria never leaving stasis. In archaea and eukarya, ribosomal expansion has been incremental and iterative, without substantial remodeling of pre-existing structures of the common core. This is best exemplified by the conserved functional centers of the ribosome, the A-site, the mRNA decoding center (DCC) of the SSU, the P-site, the peptidyl transferase center (PTC) of the LSU, the E-site, where tRNAs leave the ribosome, and the peptide exit tunnel, where the growing peptide chain leaves the ribosome [31]. However, there is considerable elaboration of rRNA sequence and structure in the transition from bacteria to eukaryotes, with eukaryote-specific expansion segments in the rRNA and additional RPs [32] (Figure 1). Therefore, most of the increase in molecular mass of eukaryotic ribosomes is contributed by long rRNA expansion segments, which form helices attached to base rRNA sequence tracts found already in bacteria and which in humans extend for up to a hundred Å (Angstrom) from the ribosomal surface. In contrast, from yeast to man, the 80 ribosomal proteins of the cytoplasmic ribosome during evolution have been remarkably conserved in sequence, structure and topological position on the ribosome. This observation suggests a persistent functional role for eukaryotic RPs, either for contributions to ribosome assembly (see below) or for contributions to fine-tune protein synthesis or both [33,34].

## 3. Genomic Organization, Transcriptional Control, Processing and Modification of Ribosomal DNA

### 3.1. Genomic Organization of Ribosomal DNA Repeats and the Nucleolar Organizer Region

**Figure 2:**
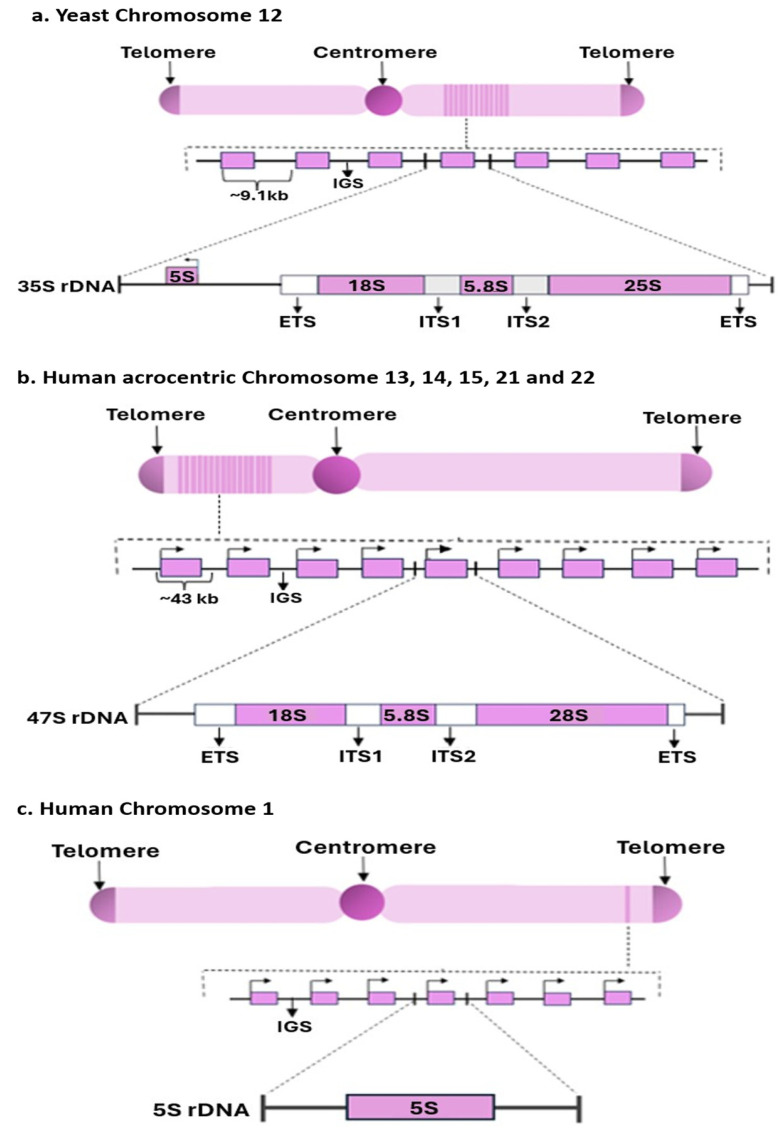
Ribosomal DNA organization in yeast and human. (**a**) In *Saccharomyces cerevisiae*, ~150 tandem rDNA repeats are clustered on chromosome XII. Each ~9.1 kb repeat contains the 35S rRNA gene, with external transcribed spacer (ETS) and internal transcribed spacer sequences (IST) and harboring the precursors of 18S, 5.8S and 25S rRNAs and the separately transcribed 5S rRNA gene, separated by intergenic spacers (IGS), harboring regulatory elements for transcription and replication. (**b**,**c**) In humans, the 47S rDNA repeats reside in large tandem arrays on the short arms of the acrocentric chromosomes 13, 14, 15, 21 and 22, whereas 5S rDNA is organized in distinct tandem arrays on chromosome 1, modified from [41].

The genomic architecture of ribosomal DNA (rDNA) reports on the evolutionary demands and solutions to the challenge of maintaining robust and adaptable ribosome function and ribosome biogenesis. Both *Saccharomyces cerevisiae* and *Homo sapiens* feature tandem arrays of rDNA repeats (Figure 2), which form the structural and functional core of the nucleolar organizer region (NOR)—a specialized subcompartment of the nucleus dedicated to ribosome biogenesis [35,36]. In yeast, all rDNA repeats are clustered on chromosome XII, with each 9.1 kb repeat encoding both the 35S rDNA gene (precursor for 18S, 5.8S, and 25S rRNAs) and the 5S rRNA gene, separated by a relatively short intergenic spacer (IGS) [37]. The IGS contains essential regulatory elements such as replication origins and RNA polymerase I promoters, ensuring high transcriptional output [38,39]. In humans, rDNA repeats, while of orthologous organization, are much larger (~43 kb each), are located on the short arms of five acrocentric chromosomes (13, 14, 15, 21, and 22) and harbor the 5S rDNA gene repeats on chromosome 1 [36,40,41]. The human IGS is significantly expanded and contains multiple regulatory elements, repetitive sequences, and embedded small nucleolar RNA (snoRNA) genes [35,42]. This structural complexity allows for nuanced regulation and tissue-specific expression of rRNA genes in a complex organism.

### 3.2. Transcriptional Control of rRNA Genes

Both yeast and humans maintain rDNA repeat number and transcriptional activity through epigenetic mechanisms, including DNA methylation, histone modification, and chromatin remodeling [43,44]. In humans, additional regulatory layers—such as p53-mediated repression and tissue-specific rDNA variants—reflect the needs of complex multicellular organisms [45,46]. Recent studies highlight that rDNA copy number, sequence variation, and expression are dynamically regulated in response to development, environmental cues, and also in disease. Transcriptomic analyses confirm that rDNA heterogeneity underpins ribosomal diversity and functional specialization [47,48,49].

rRNA gene transcription by Pol I initiates in the nucleolus, with yeast and humans employing homologous yet increasingly complex regulatory mechanisms. In yeast, the rDNA promoter consists of a core element and an upstream control element (UCE), which recruit the upstream activating factor (UAF) and core factor (CF) complexes. These, together with the Pol I-specific factor Rrn3, assemble the pre-initiation complex (PIC) and facilitate Pol I recruitment [50,51]. The nutrient-sensing TOR (target of rapamycin) pathway is central to regulating Pol I activity, linking rRNA synthesis to cellular growth conditions via phosphorylation of Rrn3 [52,53,54]. In humans, the rDNA promoter is structurally more elaborate, with additional regulatory sequences and binding sites. The selectivity factor 1 (SL1) and upstream binding factor (UBF) replace yeast UAF/CF, and the Rrn3 ortholog TIF-IA mediates Pol I recruitment [50,55]. Human rRNA transcription is regulated by a broader array of signaling pathways, including mTOR, PI3K/AKT, and MAPK, which integrate mitogenic, metabolic, and stress signals [56,57,58]. Notably, the p53 pathway represses rRNA transcription under stress or DNA damage, a regulatory layer not present in yeast [59,60]. While yeast relies primarily on TOR signaling to couple nutrient availability to rRNA synthesis, humans have evolved a more intricate network of signaling pathways to fine-tune rRNA transcription in response to diverse physiological and developmental cues [61,62]. This complexity supports the demands of multicellular life and tissue specialization.

### 3.3. Processing of rRNA Precursors

**Figure 3:**
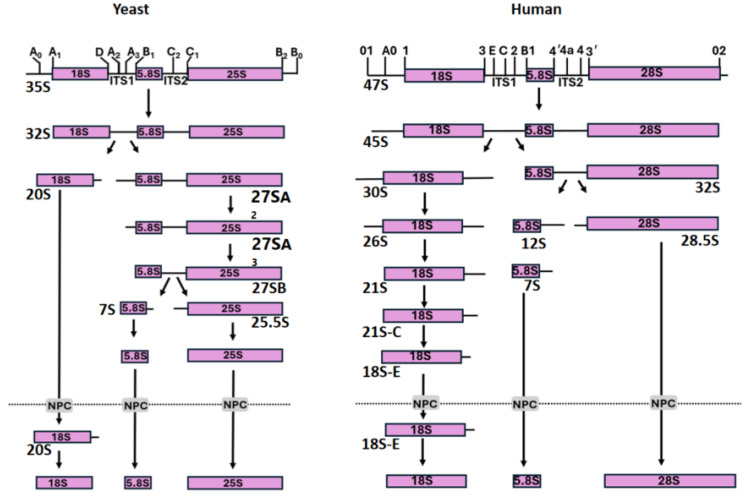
Simplified model of the rRNA processing pathway in yeast and human. This schematic summarizes pre-rRNA processing in yeast and human cells, highlighting the stepwise maturation of the 35S/47S primary transcripts into 18S, 5.8S and 25S/28S rRNAs. Major cleavage steps within the 5′ and 3′ external transcribed spacers (ETS) and internal transcribed spacers (ITS1/ITS2) are indicated, together with the principal intermediates and their sub-nuclear versus cytoplasmic localization with the transit nuclear pore complex (NPC) indicated. Processing sites are also indicated and corresponding enzymatic actions are outlined in the main text. This overview emphasizes both conserved features and species-specific branching of the pathway. Minor alternative processing pathways are not shown for clarity, modified from [66,82,83].

Following transcription by Pol I in the nucleolus, the large precursor rRNAs—35S in yeast and 47S in humans—undergo a series of processing steps to generate mature rRNAs (Figure 3). These steps are tightly coupled to ribosome assembly and involve precise endonucleolytic and exonucleolytic cleavages, guided by conserved and species-specific factors (see also below ribosome biogenesis). Early cleavage events, both in yeast and humans, are initiated by the small subunit (SSU) processome, a large ribonucleoprotein complex containing U3 snoRNA and numerous associated proteins [63,64]. This complex mediates the removal of the 5′ external transcribed spacer (5′ ETS) from the precursor rRNA. In humans, the processome is more complex, reflecting the larger precursor and greater nucleolar complexity [65,66]. Next, internal transcribed spacers (ITSs), ITS1 and ITS2, which are critical for proper, early rRNA folding, are processed. In yeast, cleavage of ITS1 is catalyzed by the RNase MRP complex, which also links rRNA processing to cell cycle regulation [67,68]. The exosome complex and exonucleases such as Xrn1 and Rat1 further process and trimm rRNA intermediates [69,70]. In humans, ITS1 processing is more intricate, involving not only RNase MRP but also additional endonucleases (e.g., NOB1, PNO1) and the exosome complex for exonucleolytic trimming [39,71,72]. The exosome and XRN2 (the human homolog of yeast Xrn1) degrade excised spacer sequences and trimm the 5′ ETS, while exconucleases RAT1/DHX37 process the 3′ ends of rRNA intermediates [73,74]. In yeast, the final stages of rRNA processing occur predominantly within the nucleolus, yielding mature 18S, 5.8S, and 25S rRNAs [33,75]. The 18S rRNA undergoes its final maturation in the cytoplasm, while 5.8S and 25S rRNAs are exported as nearly mature molecules [76,77]. In humans, late processing steps similarly produce mature 18S, 5.8S, and 28S rRNAs, with 18S rRNA final maturation occurring in the cytoplasm [78,79,80]. The increased complexity of human rRNA processing is reflected by the involvement of additional nucleases, assembly factors, and quality control checkpoints [65,66,81].

Both systems employ quality control mechanisms to ensure fidelity. In yeast, the exosome degrades misprocessed rRNAs [84,85]. In humans, the exosome is similarly involved, but additional nuclear RNA surveillance pathways provide further control [86,87,88]. Advances in cryo-EM and transcriptomics have revealed the dynamic architecture and interactions of these processing machineries [63,81,89].

#### Modification of Ribosomal RNA Nucleotides

rRNA nucleotide modification is essential for ribosome function and is predominantly guided by snoRNAs, which direct site-specific 2′-O-methylation and pseudouridylation [90,91]. In yeast, about 55 rRNA modifications are guided by snoRNAs, typically encoded within introns of ribosomal protein genes or genes for ribosome biogenesis factors [92,93]. U3 snoRNA, for example, is crucial for early processing steps and is transcribed independently of rDNA but functionally coupled to rRNA synthesis [94,95]. In humans, the repertoire of rRNA modifications is expanded to over 100, with many snoRNAs (e.g., U8, U13) embedded within rDNA IGS and co-transcribed with pre-rRNA by Pol I, ensuring synchronized production of guides and targets [42,96,97]. This enables tissue-specific and condition-specific rRNA modification patterns, supporting functional diversification of ribosomes in multicellular contexts [46,98,99]. Recent studies have linked human-specific rRNA modifications to tissue specialization, stress response, and disease, highlighting the evolutionary adaptation of the ribosome for complex cellular environments [46,97]. In conclusion, to ensure synchronized production levels of rRNA, yeast and human rRNA biogenesis share a conserved core of genomic organization, transcriptional control, processing, and modification, but the human system is characterized by greater complexity and regulatory capacity. This reflects the evolutionary pressures of multicellularity, tissue specialization, and environmental responsiveness. Advances in genomics, structural biology, and transcriptomics continue to reveal new layers of regulation and adaptation in ribosome biogenesis across eukaryotes [81,97].

## 4. Genomic Organization and Expression Regulation of Ribosomal Proteins in *Saccharomyces cerevisiae* and *Homo sapiens*

### 4.1. Genomic Organization and Evolutionary Trajectories

The study by Petibon et al. provides an excellent and comprehensive analysis of ribosomal protein gene (RPG) structure and distribution across the main kingdoms of life [100] and also includes analysis of earlier work on this topic [101,102]. Here, we focus on the comparison between the eukaryotic yeast and human systems, where the number of ribosomal proteins does not change, but where the regulation of expression of the ribosomal protein genes has become much more intricate in humans. In general, the genomic architecture and regulatory mechanisms governing ribosomal protein gene (RPG) expression have undergone significant evolutionary diversification across kingdoms. While prokaryotes maintain compact operonic arrangements optimized for rapid co-expression, eukaryotes have evolved intricate multi-layered regulatory systems that balance precision with adaptability [103,104,105,106]. Here, we report on divergent strategies employed by yeast and humans to regulate RP synthesis, including a spotlight on emerging insights into translational control mechanisms and their implications for cellular homeostasis.

Eukaryotic RP genes (RPGs) with respect to non-coding regions exhibit marked divergence from their prokaryotic counterparts, and this architecture enables sophisticated fine-tuned regulation. In *Saccharomyces cerevisiae*, RPGs average 1.5 kb with minimal intronic content, reflecting a streamlined genome optimized for rapid growth [103,104,107,108]. In contrast, human RPGs exceed 10 kb due to elongated introns and untranslated regions (UTRs), hosting platforms for alternative splicing and context-dependent regulation. For instance, alternative splicing of the *RPL22* gene generates isoforms differentially expressed in muscle and hepatocytes, fine-tuning ribosome composition to meet tissue-specific demands [105,109,110]. Archaeal genomes, while retaining short UTRs and rare introns, lack the regulatory complexity seen in eukaryotes, underscoring the evolutionary innovation of intron-mediated regulation in higher organisms [111,112,113,114].

The ribosomal protein complement on the translation-competent ribosome is invariantly 78 RPs for yeast and 79 RPs for humans. However, gene duplication events have further shaped the evolution of RP expression strategies, with yeast presenting 137 RPGs, with 118 RPGs transcribed from duplicated, paralogous genes and with humans presenting ~90 RPGs [33,103,104,107,115]. However, numerical expansion alone does not explain eukaryotic regulatory complexity. Sub-functionalization of paralogous ribosomal proteins, such as yeast rpL22a/eL22 and rpL22b/eL22, enables specialized roles in stress adaptation—rpL22a/eL22 supports translation during amino acid deprivation, while rpL22b/eL22 is critical for oxidative stress responses [116,117]. This functional diversification in eukaryota contrasts markedly with bacterial operons, where polycistronic transcription ensures stoichiometric RP production but limits regulatory flexibility [103]. Comparative genomics studies reveal that human RPGs have acquired lineage-specific exons through retrotransposition events, enabling novel regulatory interfaces with RNA-binding proteins like HNRNPK [118,119].

### 4.2. Transcriptional Regulation Networks

Transcriptional control of RPGs diverges markedly between yeast and humans, reflecting distinct evolutionary pressures. In yeast, the Rap1-Fhl1-Ifh1 complex binds conserved promoter motifs, enabling rapid nutrient-responsive regulation [120,121]. This system permits near-instantaneous transcriptional activation upon glucose availability, with chromatin remodelers like the RSC (Remodels the Structure of Chromatin) complex modulating promoter accessibility within minutes of metabolic shifts [122,123]. Recent single-cell analyses reveal that yeast RPG transcription directly couples to cellular ATP levels, with nucleosome repositioning acting as a metabolic sensor [124,125,126]. During diauxic shift, the Hda1 histone deacetylase represses RPG promoters by stabilizing nucleosomes over Rap1-binding sites, demonstrating how chromatin dynamics fine-tune transcriptional output [127,128]. Human RPGs employ a more decentralized regulatory architecture. MYC, SP1, and GABP transcription factors act through dispersed enhancer elements, integrating growth signals with epigenetic modifiers such as histone acetyltransferases [129,130]. Notably, studies using CRISPR-mediated enhancer deletion have shown that human RPGs rely on redundant regulatory elements, ensuring robustness against genetic or environmental perturbations [131,132]. For example, the knockdown of the ribosomal Protein eL29 in mammalian cells leads to significant changes in the HEK293 cells. The deficiency of eL29 is associated with altered expression of a number of genes that are targets of p53 or c-Myc [133]. Interestingly, ribosomal protein mRNAs, such as ecoded rpL26/uL24 and rpS27/eS27, are exclusively upregulated in distinct cancers such as breast and thyroid, while the level of RPL21 mRNA is decreased in breast and uterine cancers [134,135].

### 4.3. Translational Control Mechanisms of RP mRNAS

Translational regulation in *S. cerevisiae* operates through three primary mechanisms. First, codon optimality—particularly near start sites—modulates initiation rates. The term codon optimality encompasses both optimal and strategically suboptimal codon usage. For example, during stress, suboptimal codons in RP mRNAs slow ribosome loading, preventing resource-intensive elongation until conditions improve [136,137,138]. Second, autoregulatory feedback ensures stoichiometric balance, as many ribosomal proteins regulate their own translation by binding to sequences within their mRNA that structurally mimic the ribosomal RNA binding sites they recognize during ribosome assembly. When the concentration of the ribosomal protein exceeds the available rRNA, surplus protein binds its own mRNA, blocking further translation and maintaining homeostasis [139]. For example, excess rpL4/uL4 binds its mRNA’s 5′ UTR, occluding the Kozak sequence and blocking initiation of translation [140,141]. Third, mRNA structural switches enable context-specific translation. For example, the *RPS28* mRNA contains thermally unstable hairpins that unfold at elevated temperatures, exposing internal ribosome entry sites (IRES) for heat shock-specific translation [142,143,144]. A recent review by Chekulaeva (2024) identified 12 yeast RP mRNAs with similar thermosensitive structures, suggesting a widespread mechanism for stress adaptation [145]. Furthermore, recent work in Diamond–Blackfan anemia patients has identified mutations in rpS19/eS19 translational control elements, linking dysregulated RP synthesis rather than mutations in the ribosomal protein gene to erythroid failure [146,147].

Mammalian RP synthesis integrates additional control mechanisms. The 5′ terminal oligopyrimidine (TOP) motif, present in 80% of human RP mRNAs, recruits the RNA-binding protein Larp1. Under nutrient deprivation, Larp1 sequesters TOP mRNAs in cytoplasmic granules, but mTORC1 activation phosphorylates Larp1, releasing transcripts for polysome loading [148]. This system allows precise coordination with metabolic status. Additionally, collision surveillance mechanisms prevent toxic RP aggregation: ZNF598 (zing finger protein 598) ubiquitinates ribosomes stalled on RP mRNAs, marking them for NoGo-mediated decay (NGD) [149,150,151]. This pathway is essential for maintaining proteostasis in neural stem cells, where RP overproduction disrupts differentiation [152]. IRES-dependent initiation provides another layer of regulation. Over 47 human RP mRNAs, including *RPL10A*, contain functional IRES elements enabling translation during mitosis or oncogenic stress when cap-dependent initiation (see below) is suppressed [153,154]. Remarkably, *RPL23A* IRES activity increases 12-fold during DNA damage, ensuring ribosome function despite transcriptional shutdown [155]. Recent work using ribosome profiling in glioblastoma cells showed that IRES-mediated RP translation sustains tumor growth under hypoxic conditions, highlighting its clinical relevance [105,156].

Recent advances have uncovered novel regulatory layers. For example, eS26-deficient ribosomes preferentially translate mRNAs with weak Kozak sequences during amino acid starvation, prioritizing stress-response proteins over RPs [157,158]. In another example, selective translation depends on phosphorylation of uS10 by stress-responsive GCN2 kinase, which alters ribosome subunit stability. Phase separation also plays a critical role: under oxidative stress, G3BP1/2 proteins sequester RP mRNAs into condensates, dynamically releasing them upon redox normalization [159,160]. Super-resolution microscopy has captured these condensates, which exclude active ribosomes, suggesting they function as temporary mRNA storage compartments [161,162]. Furthermore, tRNA reprogramming via METTL6-mediated methylation of tRNA-Leu (CAA) enhances decoding of *RPL5* mRNA codons, creating a feedforward loop that amplifies RP synthesis during lymphocyte proliferation [163]. This latter epitranscriptomic regulation mechanism is conserved in mammals but absent in yeast, reflecting evolutionary divergence in translational control strategies.

### 4.4. Nuclear Import Pathways

RP trafficking mechanisms highlight species-specific adaptations. Yeast employs Syo1 symportin to co-import Rpl5/Rpl11 with 5S rRNA, ensuring coordinated subunit assembly [76]. Mutations in Syo1 disrupt 60S subunit maturation, leading to nucleolar fragmentation—a phenotype rescued by artificial tethering of Rpl5 to 5S rRNA [164,165]. In humans, importin homologs (RanBP5/7) mediate > 80% of RP transport using bipartite nuclear localization signals (NLS). Real-time imaging studies [166] reveal the cytoplasmic half-life of RPs prior to nuclear import of <3 min, necessitating chaperones to prevent aggregation [167,168]. Dysregulation of these pathways underlies nucleolar stress responses, as delayed delivery of ribosomal proteins to form 40S subunits ready for export triggers p53 activation and cell cycle arrest [169,170,171]; Intriguingly, cancer-associated mutations in *rpL5* NLS motifs disrupt nuclear import, activating p53-mediated senescence—a finding with implications for targeted therapies [172].

So far, the observations on the regulation of rRNA and ribosomal protein expression underscore the demand for an ordered supply of these ribosomal components for ribosome biogenesis, a major cellular nexus integrating growth signals, stress adaptation, and developmental checkpoints.

## 5. Eukaryotic Ribosome Biogenesis

### 5.1. The Energy Cost of Ribosome Biogenesis

Ribosome biogenesis represents one of the most energy-intensive processes in eukaryotic cells, both with respect to the number of ribosomes produced and resources consumed.

Human cells face similar challenges, with ribosome biogenesis consuming ~80% of cellular energy in rapidly dividing cells [173], though their larger ribonucleoprotein complexes and additional regulatory layers introduce greater complexity [174].

In rapidly proliferating cells, ribosome biogenesis proceeds at very high but comparable orders of magnitude in yeast and mammals. Based on estimates from budding yeast, a typical cell produces approximately 2 × 10^5^ ribosomes over a ~100 min generation time, corresponding to roughly 2000 mature ribosomes per minute per cell. In human HeLa cells, about 4 × 10^6^ ribosomes are synthesized over a ~1200 min division cycle, yielding an average of ~3300 ribosomes per minute per cell [175,176,177]. These values are generalized, approximate numbers derived under specific growth conditions and should be interpreted as order-of-magnitude estimates rather than universal constants, illustrating that although yeast and mammalian cells operate on different temporal and volumetric scales, their ribosome production rates per minute are broadly similar, with mammalian cells in this example exhibiting a modestly higher average output [178].

Ribosome biogenesis is one of the most energy-demanding biosynthetic programs in eukaryotic cells, with a large fraction of transcription and translation committed to producing ribosomal components. In budding yeast, about 60% of total nuclear transcription is devoted to rRNA synthesis by RNA polymerase I, and on the order of 50% of RNA polymerase II transcription and the majority of mRNA splicing capacity are devoted to ribosomal protein mRNAs, reflecting that ribosomal proteins constitute roughly 30% of total cellular protein mass and about half of all protein molecules [178]. Mammalian cells show a similar prioritization: in rapidly dividing human cells, ribosome biogenesis has been estimated to consume a major fraction—up to 80%—of the cellular energy budget [173] and is accompanied by high rRNA transcription rates, large ribosomal protein output, and the assembly of millions of ribosomes per cell, all coordinated through additional regulatory and quality-control layers that further increase the energetic cost of maintaining an adequate ribosome supply.

In general, evolutionary adaptations optimize energy efficiency through tight regulatory networks. In yeast, the ribosome assembly stress response (RASTR) monitors unassembled RPs, triggering Hsf1-mediated chaperone production and Ifh1 sequestration to balance biosynthesis with proteostasis [179]. This prevents wasteful overproduction by linking RP gene transcription to assembly capacity [176,179]. Human cells employ analogous quality control systems, where unassembled RPs activate p53 pathways to limit unnecessary synthesis [174,179]. Both systems minimize energy expenditure by rapidly adjusting production rates of ribosomal proteins and rRNA through transcription factor regulation [91,176,177,179].

### 5.2. Nucleolar Initiation: Transcription and Phase-Separated Compartmentalization

**Figure 4:**
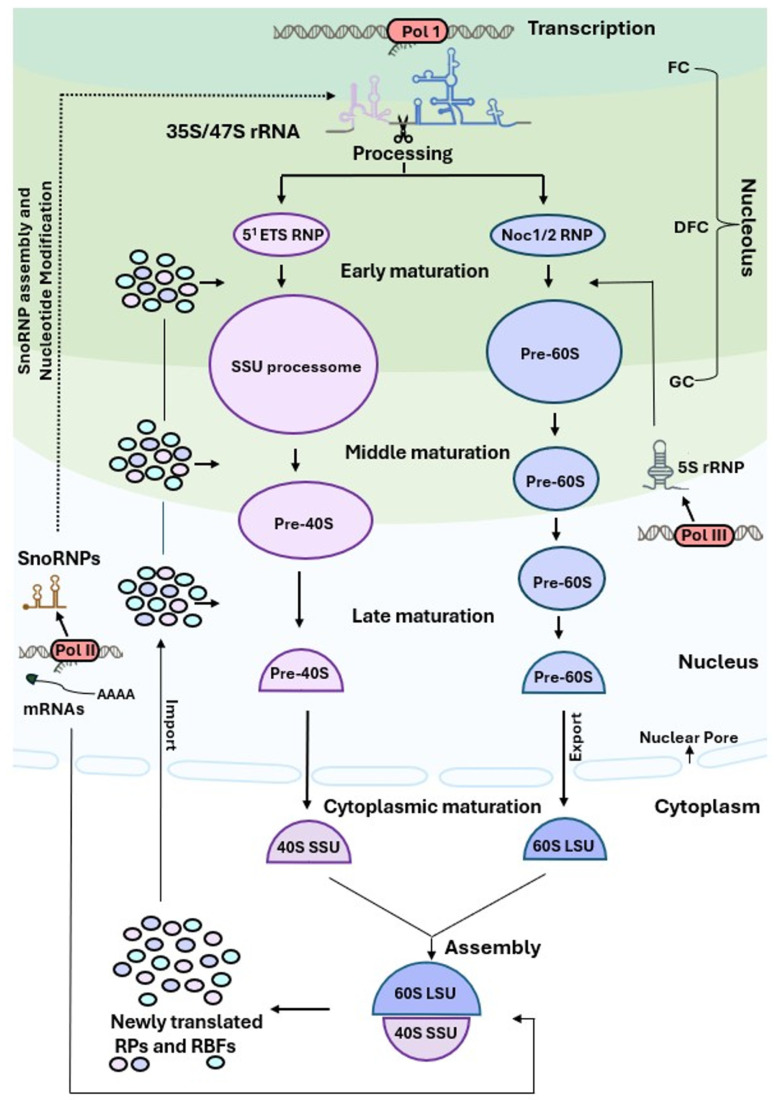
Ribosome biogenesis: dynamic successive shaping of ribosomal subunits from nucleolus to cytoplasm. Ribosome biogenesis is a successive, compartmentalized remodeling process in which nascent preribosomal particles are progressively shaped into translation-competent ribosomal subunits. Pol I–driven transcription of precursor rRNA in the nucleolus initiates co-transcriptional folding, processing, and association with assembly factors and ribosomal proteins, giving rise to early precursors of the small (SSU/40S) and large (LSU/60S) subunits. These immature particles undergo stepwise structural remodeling and quality control in early, middle and late maturation steps, as they transit from the nucleolus through the nucleoplasm, acquiring their functional architecture before export through the nuclear pore complex into the cytoplasm. In the cytoplasm, final maturation steps complete the assembly of 40S and 60S subunits, which are then ready to join and form 80S ribosomes capable of mRNA-directed protein synthesis, modified from authors [186,187].

Ribosome biogenesis in eukaryotes is a highly coordinated and dynamic process essential for cellular viability, occurring predominantly within the specialized nuclear compartment known as the nucleolus (Figure 4). Unlike prokaryotic systems, where ribosome assembly factors are primarily required under stress conditions, eukaryotic ribosome biogenesis necessitates a sophisticated machinery comprising over 200 essential factors, the ribosome biogenesis factors (RBGs) [32,177,180,181]. These factors guide every stage of assembly, from the transcription of ribosomal RNA (rRNA) in the nucleolus to the final maturation of ribosomal subunits in the cytoplasm. This intricate process ensures the precise folding, modification, and stoichiometric incorporation of ribosomal proteins (RPs) into nascent ribosomal subunits, while quality control mechanisms safeguard the functional integrity of the mature ribosome [31].

The nucleolus, a membrane-less organelle, serves as the primary site for ribosome biogenesis. Its formation is driven by liquid-liquid phase separation (LLPS), a process that concentrates rRNA, ribosomal proteins, and assembly factors while excluding nonspecific macromolecules. Through LLPS, the nucleolus is further organized into distinct, coexisting liquid-like subcompartments that constitute functionally specialized domains, thereby spatially separating and coordinating individual steps of ribosome biogenesis—such as rRNA transcription, processing, and ribosomal subunit assembly—within a single, dynamic organelle [182].

In humans, RNA polymerase I (Pol I) transcribes the 47S rRNA precursor, which contains the 18S, 5.8S, and 28S rRNA sequences flanked by external and internal transcribed spacers (ETS/ITS) (Figure 2, Figure 3 and Figure 4). The extended 5′ ETS in humans enhances nucleolar LLPS by interacting with scaffolding proteins like nucleophosmin (NPM1), which stabilize phase-separated condensates [183,184]. These condensates organize the nucleolus into sub compartments. These are the fibrillar centers (FCs), the sites of rDNA transcription by Pol I, the dense fibrillar components (DFC), the regions where early rRNA processing and chemical modifications occur and granular components (GC), the zones for late assembly steps and pre-ribosome export [182,185].

In yeast, the shorter 5′ ETS limits phase separation complexity compared to humans, yet both systems rely on LLPS to spatially and temporally coordinate rRNA processing. The transcription of rRNA is coupled to its initial folding, as the emerging transcript interacts with small nucleolar RNAs (snoRNAs) and associated proteins. These interactions prevent misfolding and guide chemical modifications, such as 2′-O-methylation and pseudouridylation, which are critical for rRNA stability and ribosome function (Figure 4) [183]. Recent studies using fluorescence recovery after photobleaching (FRAP) and fluorescence loss in photobleaching (FLIP) have demonstrated that nucleolar components like fibrillarin and nucleolin rapidly exchange between the nucleolus and nucleoplasm, with residence times as short as 50–80 s [182,188]. This dynamic exchange suggests that assembly factors cycle through the nucleolus during successive rounds of ribosome biogenesis, likely regulated by phosphorylation-dependent activation states [188,189].

### 5.3. rRNA Processing in the Context of SSU Processome Formation and LSU Pre-60S Particle Formation

Following transcription, the human 47S rRNA undergoes extensive processing to yield mature 18S (40S subunit), 5.8S, and 28S (60S subunit) rRNAs (Figure 3). The small subunit (SSU) processome (formerly termed the 90S pre-ribosome) plays an exclusive role in 40S subunit biogenesis, resolving historical ambiguities about its involvement in 60S assembly [82,183,190]. This massive ribonucleoprotein (RNP) complex scaffolds the 18S rRNA domains via U3 snoRNA-mediated base pairing, while excluding LSU components [82]. One key step within SSU processome maturation is a series of endonucleolytic cleavages in the 5′ ETS and ITS1, where the 35S/47S pre-rRNA is sequentially processed at sites A0, A1, and A2. These early cleavages are carried out within the SSU processome, with the PIN-domain endonuclease Utp24 providing the catalytic activity at A1 and A2 and contributing to A0 processing, while factors such as Rcl1 are particularly important for efficient cleavage at A2. Cleavage at A2 in ITS1 functionally separates the 18S rRNA precursor (20S pre-rRNA) from the precursors of the 5.8S and 25S rRNAs, thereby partitioning the small-subunit pathway from large-subunit rRNA maturation for further downstream processing steps [191].

Further steps are helicase activities, exemplified by Dhr1m, which remodels U3 snoRNA-18S hybrids, facilitating the transition from the SSU processome to pre-40S particles [192]. Quality control is executed by the nuclear exosome function. An exosome is a multi-protein 3′ → 5′ exoribonuclease complex that processes and degrades RNA. Exosome activity particularly targets pre-rRNA and various by-products or defective intermediates thereof, and drives structural remodeling of the 90S particle into a primordial pre-40S, by preventing re-annealing or accumulation of RNA debris that would stall maturation [190].

The SSU processome’s protein composition—51 assembly factors and 18 RPs—ensures architectural specificity. Cryo-EM studies reveal that the 90S structure assembles in a stepwise manner as the pre-rRNA transcript serves as a platform for assembly of proteins, with the 5′ ETS acting as a seed for recruiting modules like UTP-A, UTP-B, and the U3 snoRNP [183,190]. On the level of ribosomal RNA, the transcription of helix 44 in the 18S rRNA triggers a dramatic compaction of the 90S particle, enabling the incorporation of later factors like Utp20 and Bms1-Rcl1, which establish long-range connections between ribosomal subdomains [183]. Human pre-40S particles retain factors like Enp1 and Ltv1 to coordinate nuclear export, whereas yeast pre-40S complexes are simpler, reflecting reduced regulatory demands [190]. Further remodeling forceS are nucleolar DEAH-box ATPases, which restructure the 90S processome by coupling ATP hydrolysis to local unwinding of RNA and disruption of RNA–protein contacts, enabling stepwise reconfiguration into export-competent pre-40S particles. These ATPases displace U3 snoRNA and associated factors from the pre-rRNA, promote compaction of the 18S region, and help release early assembly factors so that late biogenesis proteins and ribosomal proteins can bind (Figure 4). By enforcing directionality and quality control of these remodeling events in the nucleolus and nucleoplasm, ATPases drive irreversible progression of small-subunit maturation toward the cytoplasmic stages [193,194,195].

The biogenesis of the pre-60S ribosomal subunit is also a meticulously orchestrated process involving sequential rRNA processing, structural remodeling, and quality control checkpoints. Following the initial cleavage of the 47S pre-rRNA that separates the small and large subunit pathways, the 27S/32 S pre-rRNA intermediate undergoes a series of maturation steps to generate the 5.8S and 25S/28S rRNAs. Analogous to the SSU processome, which is dedicated to 40S assembly, the pre-60S particle traverses distinct macromolecular complex states during maturation [186,196,197,198]. Central to this process is the internal transcribed spacer 2 (ITS2), which is excised through endonucleolytic cleavage at site C2 by the Las1-Grc3 nuclease complex, a step essential for liberating the 5.8S-28S rRNA duplex [199]. Subsequent exonucleolytic trimming by the exosome and Xrn2 ensures precise rRNA termini, while RNA helicases such as Dbp10 and Mak16 restructure rRNA domains to facilitate ribosomal protein (RP) incorporation [200,201,202]. Cryo-EM studies have illuminated this dynamic architecture of pre-60S particles. Early nucleolar stages involve the hierarchical recruitment of factors like Noc1-Noc2, which chaperone the 5S RNP integration via the Rpf2-Rrs1 scaffold, and the PeBoW complex (Pes1-Bop1-WDR12), which coordinates ITS2 processing [190,203,204]. The transition to more advanced nucleoplasmic pre-60S particles is marked by the departure of early factors and the arrival of export adaptors like Nmd3 and Tif6, which prevent unscheduled premature subunit joining of immature subunits [205]. After nuclear export, structural rearrangements during cytoplasmic maturation involve ATPases such as Drg1, which displaces Nmd3, and the zinc-finger protein Rei1, which collaborates with chaperone Jjj1 to facilitate the later cytosolic 60S polypeptide exit tunnel [206]. These steps are tightly coupled to the removal of remaining assembly factors, ensuring only structurally intact subunits engage in translation.

While the formation of both ribosomal subunits has to integrate dynamic and flexible steps, there are steps that command irreversible architectural specificity. ATP-dependent helicases (e.g., Dhr1, Dbp10) and AAA+ ATPases (e.g., MDN1/Rea1, Rix7/NVL2) enforce irreversible structural transitions. MDN1, for instance, extracts assembly factors like NLE1/Rsa4 from pre-60S particles via its MIDAS domain, a mechanism conserved from yeast to humans [207,208]. Structural studies at 2.3 Å resolution show that human MDN1-NLE1 interactions mirror yeast Rea1-Rsa4 contacts, underscoring evolutionary conservation [208]. On the other hand, processing pathways in series allow maturation of distinct rRNA domains, enhancing efficiency without compromising fidelity. For example, while the 5′ domain of 18S rRNA folds co-transcriptionally, the 3′ major domain undergoes remodeling in later stages, guided by factors like Utp30 and Enp1 [183].

Quality surveillance mechanisms are critical for maintaining ribosomal fidelity. Aberrant pre-60S particles are targeted for degradation via the ubiquitin-proteasome system, as exemplified by the E3 ligase Tom1, which marks misfolded RPs for disposal [87,209]. Additionally, the RNA exosome selectively degrades misprocessed rRNA in coordination with the TRAMP complex, while the Arx1-Alb1 heterodimer acts as a chaperone to prevent premature rRNA folding [210]. Recent studies highlight species-specific adaptations, such as the human-specific ATPase ABCE1, which facilitates cytoplasmic recycling of assembly factors, and the yeast-specific Bud20, which ensures proper 5S RNP orientation [206]. These insights underscore the pre-60S assembly pathway as a conserved yet adaptable framework, balancing structural precision with regulatory flexibility to maintain proteome integrity.

### 5.4. Hierarchical Assembly of Ribosomal Proteins

Ribosomal proteins associate with both the 90S processome and emerging pre-60S particles, respectively, in a highly ordered, hierarchical manner that tracks rRNA transcription and folding. Early-binding proteins recognize nascent rRNA domains and stabilize initial helices and long-range contacts, creating protein–RNA “nucleation centers” that define the overall topology of future 40S and 60S subunits, respectively (Figure 4). These early proteins are often essential; their absence destabilizes very early preribosomes and triggers turnover, emphasizing their role in establishing the basic architectural scaffold [211,212,213,214].

Middle-acting ribosomal proteins dock onto pre-formed rRNA–protein neighborhoods, bridging domains and consolidating functional regions such as the decoding center or peptidyl transferase region. Their binding frequently depends on specific early proteins and correct rRNA processing steps, so maturation pauses if these prerequisites are not met, enforcing quality control [215].

Late-associating proteins join on more mature pre-40S and pre-60S intermediates, often in the nucleoplasm or cytoplasm, to refine active sites, intersubunit interfaces, and factor-binding surfaces. These late proteins can be conditionally required, but they tune translational accuracy and subunit stability, and their incorporation is tightly coupled to the release of assembly factors and export competence [213].

Overall, directionality arises from dependency networks: early proteins and rRNA cleavages create binding platforms for middle proteins, which in turn enable late proteins, while incorrect or incomplete intermediates are selectively degraded. This stepwise program ensures that the correct three-dimensional architecture of both 40S and 60S subunits emerges only when preceding structural checkpoints are satisfied [189,216].

### 5.5. Quality Control Checkpoints

Quality control mechanisms accompany the dynamic structuring of the subunits and are integrated throughout ribosome biogenesis to ensure only functional subunits proceed to translation. Key checkpoints include first, structural surveillance, where complexes like Ebp2-Rrs1 monitor the mobility of the L1 stalk in pre-60S particles, ensuring proper rRNA folding, second, functional testing, where the peptidyl transferase center (PTC) and decoding site are interrogated in late nucleoplasmic stages, and third, kinetic proofreading, exemplified by Rio2 kinase activity on late pre-40S subunits, which blocks translation initiation until cytoplasmic maturation completes [87]. Errors in ribosome biogenesis trigger degradation by exonucleases (XRN2, DIS3) or ubiquitin-proteasome targeting [87,91,217]. Bypassing these checkpoints results in misassembled ribosomes with altered translation fidelity, linked to diseases like Diamond–Blackfan anemia. For instance, mutations or haploinsufficiency of ribosomal protein RPS19 impair 40S ribosomal subunit maturation by disrupting specific steps in 18S rRNA processing, which in turn alters translational output and leads to reduced proliferation and increased apoptosis of erythroid progenitor cells—a key pathogenic feature of Diamond–Blackfan anemia (DBA) [146,218,219,220].

### 5.6. Nuclear Export of Ribosomal Subunits

Pre-ribosomal subunit particles represent a major substrate for nucleocytoplasmic transport: with an estimated export of roughly 4000 ribosomal subunits per minute across yeast nuclear pores into the cytoplasm, i.e., 2000 cytoplasmic ribosomes in the cytoplasm. A growing mammalian cell exports on the order of 7000 ribosomal subunits per minute, i.e., 3500 complete ribosomes can be assembled in the cytoplasm. This reflects’ both larger cell size and higher nuclear pore number as well as more complex assembly and trafficking pathways [174,177,221,222].

In *Saccharomyces cerevisiae*, export of ribosomal subunits is a coordinated process that couples quality control to nucleocytoplasmic transport. The 60S subunit exits the nucleus via the Crm1 exportin bound to the adaptor Nmd3, which binds nascent large subunits and provides the leucine-rich nuclear export signal required for Crm1-dependent passage through nuclear pore complexes. Structural and cell biological studies have shown that pre-60S particles engage multiple export receptors, including Nmd3–Crm1, Mex67–Mtr2, and Arx1, to traverse nuclear pores efficiently, but the precise export efficiency and transit time per particle remain incompletely defined in vivo [223]. In contrast, the 40S subunit relies primarily on the Crm1 export pathway mediated by the adaptor protein Ltv1 and export factors such as Rio2 and Dim1, which remain associated until late cytoplasmic maturation. The assembly states of 40S-bound factors determine export competency, with incomplete pre-40S particles retained in the nucleus by quality control mechanisms that prevent premature release [193,194,224].

Human cells maintain the conserved Crm1–NMD3 axis for 60S export but exhibit redundancy through inclusion of Exportin 5 (Exp5), which directly engages rRNA helix 89. This metazoan-specific pathway supplements Crm1-exported subunits and implements an additional layer of quality control by sensing conformational maturation of the central protuberance. Redundant binding of Crm1 and Exp5 allows competitive export regulation, providing an error-correction mechanism absent in yeast. For 40S subunits, human cells evolved the PDCD2L adaptor complex that bridges CRM1 to rRNA helix 33 and interacts transiently with RIOK2, improving export efficiency in multicellular contexts. PDCD2L-dependent export thereby constitutes a metazoan innovation ensuring the selective passage of properly assembled pre-40S particles. Recent studies emphasize that nuclear pore architecture and dynamic competition between export receptors generate a selective filter restricting immature subunits from entering the cytoplasm [225].

Following nuclear export, ribosomal subunits undergo cytoplasmic maturation events that finalize their translational competence. In yeast, Nmd3 remains bound to the intersubunit interface of cytoplasmic 60S ribosomes until its release is triggered by sequential actions of Lsg1 and the ATPase Rli1/ABCE1. These GTPase- and ATPase-driven remodeling steps not only liberate Nmd3 but also validate ribosome integrity through conformational proofreading [206,226,227,228]. Similarly, pre-40S particles require cytoplasmic remodeling through Fap7 and Rio1, which remove remaining biogenesis factors and test the decoding-center function before the subunit can engage in translation initiation [229]. This dual checkpoint strategy ensures that defective or immature 40S and 60S subunits cannot participate in elongation cycles.

In human cells, the cytoplasmic maturation landscape is more complex. Auxiliary factors such as PES1, BRX1, and SBDS cooperate with NMD3 to maintain export intermediates in a translation-incompetent state until proper rRNA folding and ribosomal protein accommodation are achieved [230]. Structural analyses reveal that these metazoan-specific components induce large conformational rearrangements, masking functional centers, including the peptidyl transferase site and intersubunit bridges. Recent single-molecule data further indicate that NMD3 participates in active quality surveillance by recruiting protein kinases and ubiquitin ligases that label defective subunits for degradation, integrating export fidelity with cytoplasmic ribosome quality control [226,227,230].

The conserved Nmd3 family represents a central guardian of ribosomal subunit integrity, coordinating maturation, export, and surveillance across eukaryotes. Comparative analyses between yeast and human systems illustrate evolutionary diversification: yeast employs streamlined transport optimized for rapid proliferation, while human cells deploy multilayered regulatory networks involving redundant export factors, specialized adaptors such as PDCD2L, and cytoplasmic checkpoints. These innovations reflect the growing need for translational precision in complex multicellular organisms, where ribosomal homeostasis contributes directly to proteome quality and cellular health. Current models underline the intricate interplay between export factors, maturation enzymes, and post-export quality control mechanisms as an essential determinant of ribosome function and fidelity.

## 6. Ribosome Function in Protein Synthesis: Translation of mRNA

For the non-specialist reader, we provide first a short overview of protein synthesis and then present the individual steps of translation in more detail. In the process of translation, the ribosome converts the three-letter nucleotide code of messenger RNA (mRNA) into functional protein through a tightly regulated sequence of events. The ribosome’s three main functional sites—A site (decoding by acceptance of cognate aatRNA), P site (peptide bond synthesis), and E site (exit site of tRNA)—orchestrate this molecular choreography: each codon triggers a round of aa tRNA delivery, peptide bond formation, and spatial repositioning in a 5′ → 3′ march along the mRNA. The process concludes with termination, where release factors (eRF1/eRF3) detect stop codons (UAA, UAG, UGA) and catalyze polypeptide release [231,232].

Protein synthesis begins with translation initiation, during which the small 40S ribosomal subunit—assisted by multiple initiation factors—binds to the mRNA’s 5′ cap and scans for the start codon (AUG), a checkpoint requiring dynamic mRNA structure remodeling [233]. Upon start codon recognition, the initiator tRNA (tRNA_i_^Met^) is uniquely positioned in the P-site—unlike all subsequent tRNAs, which first enter the A-site—allowing the large 60S subunit to join and form the active 80S ribosome. During elongation, incoming aminoacyl tRNAs decode successive codons at the A-site, while peptide bond formation in the P-site extends the nascent chain. When a stop codon reaches the A-site, release factors catalyze hydrolysis of the peptidyl-tRNA bond, leading to release of the newly synthesized protein and subsequent ribosome disassembly and recycling of the subunits for a new round of translation.

### 6.1. Translation Initiation

**Figure 5:**
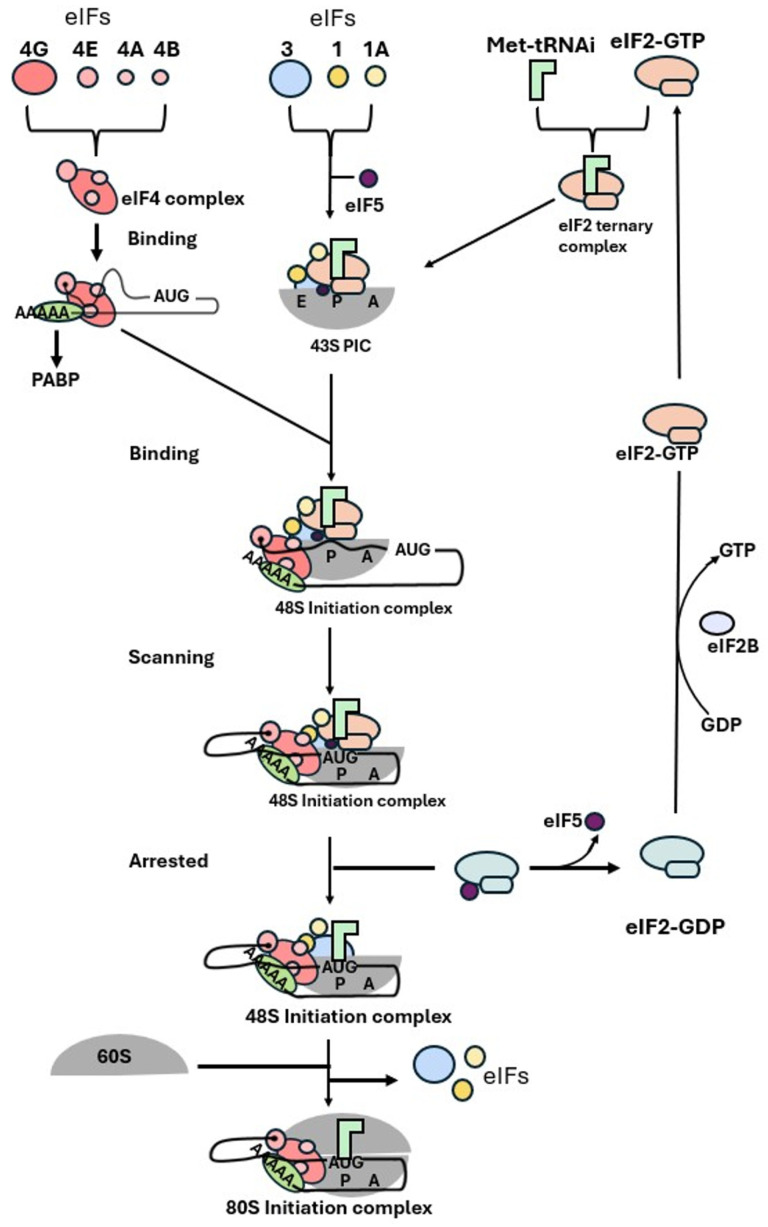
Concerted pathway of eukaryotic cap-dependent translation initiation leading to a translation-competent 80S ribosome. The figure illustrates the sequential steps leading from mRNA recognition to the assembly of a translation-competent 80S ribosome. Translation initiation begins with the selection of an mRNA by the eIF4F complex, which binds the 5′ cap and unwinds secondary structures in the 5′ untranslated region through the helicase activity of eIF4A. The 43S preinitiation complex (PIC), consisting of the 40S ribosomal subunit, eIF1, eIF1A, eIF3, eIF5, and a ternary complex of eIF2–GTP–Met-tRNA^i^, is recruited to the mRNA via interactions with eIF4F. Productive binding leads to accommodation of the 48S PIC on the 5′ end, enabling scanning along the mRNA until recognition of the AUG start codon. Base-pairing between Met-tRNA^i^ and the AUG codon stabilizes a closed conformation of the 48S complex, which then promotes recruitment of the 60S subunit to form the 80S initiation complex. This joining step is facilitated by the GTPase activity of eIF5B, culminating in a ribosome with Met-tRNAi positioned in the P site and an empty A site ready to accept the next aminoacyl-tRNA for elongation. Further mechanistic details and regulatory aspects are described in the main text, modified from [232,235].

Canonical eukaryotic translation initiation proceeds through a series of ribonucleoprotein assembly steps, in which the ordered formation and remodeling of preinitiation complexes on the 40S subunit and the mRNA culminate in joining with the 60 S subunit to form the translation-competent 80S ribosome at the start codon (Figure 5). The sequence and interdependence of these complexes ensure accurate start codon selection and directional scanning, while allowing multiple layers of regulation via initiation factors and 5′ UTR features [234].

Initiation begins with mRNA activation at the 5′ end. The cap-binding protein eIF4E recognizes the 7-methylguanosine cap, the poly(A) tail is bound by poly(A)-binding protein (PABP), and the large scaffold eIF4G bridges these elements to allow a closed-loop mRNP structure (Figure 5). Within this complex, the RNA helicase eIF4A, assisted by eIF4B and eIF4H, unwinds secondary structures in the 5′ UTR, producing an “activated” mRNA that is competent for ribosome recruitment. The activated, closed-loop mRNA provides the platform onto which the small ribosomal subunit–bound initiation machinery will be loaded [234].

In parallel, the 43S preinitiation complex (43S PIC) forms on the small (40S) ribosomal subunit. The free 40S subunit, typically recycled from a previous round of translation, associates with the multifactor scaffold eIF3 and the factors eIF1 and eIF1A. Independently, the ternary complex (TC) is assembled, consisting of eIF2 bound to GTP and initiator Met-tRNAi; together with eIF5, this TC is recruited to the 40S–eIF3–eIF1–eIF1A complex to yield a fully competent 43S PIC. The TC is central because it delivers Met-tRNAi in a conformation poised to engage the P site, but eIF1 and eIF1A maintain an open, scanning-competent state that prevents premature accommodation at non-optimal codons [234,236,237].

Evidence from biochemical and structural work supports the model that eIF4F at the cap and PABP at the poly(A) tail, bridged by eIF4G, form an activated, closed-loop mRNP that directly promotes recruitment of the 43S/40S preinitiation complex (PIC) via eIF4G–eIF3 and eIF4G–40S contacts [232]. Toeprinting in yeast extracts shows that a closed loop can be detected already at the next step, the 48S stage, before 60S joining, indicating that small-subunit–bound initiation complexes dock onto an mRNP that is already in a closed conformation. Mechanistically, eIF4A within eIF4F and an additional eIF4A at the mRNA entry site cooperate with eIF4B/H to unwind 5′ UTR structures and feed the mRNA into the 40S channel, thereby converting the closed-loop mRNP into an “activated” template competent for subsequent scanning [238,239]. Formation of the 48S complex on mRNA uses eIF3 as the principal bridge between the 43S PIC and the activated mRNP, functionally coupling the small subunit–bound factors to the eIF4F complex at the cap. Through these interactions, the 43S complex is recruited to the 5′ end of the mRNA, generating the 48S preinitiation complex, in which the 40S subunit, loaded with the TC and associated initiation factors, is positioned near the cap. The 48S complex then scans the 5′ UTR in the 5′ → 3′ direction, with eIF4A-driven unwinding and the geometry of the 40S–factor ensemble coordinating processive movement along the RNA [234].

Start codon recognition is marked by a conformational transition within the 48S complex. When a suitable AUG in an appropriate nucleotide context is encountered, the codon–anticodon pairing with Met-tRNAi in the P site induces closure of the 40S head and tighter mRNA engagement, converting the scanning-competent “open” 48S into a “closed” conformation. In this state, eIF1 is displaced from its position near the P site, eIF1A and eIF3 reorganize, and the Met-tRNAi becomes stably accommodated, solidifying start site selection. GTP hydrolysis on eIF2, stimulated by eIF5, follows AUG recognition and commits the complex to initiation by promoting release of eIF2–GDP and associated factors, thereby locking in the chosen start codon and limiting further scanning.

The 60S subunit also has to undergo final maturation steps to achieve competence for subunit joining. In particular, cytoplasmic maturation of the 60S ribosomal subunit requires structural reconfiguration mediated by specific, late ribosomal proteins and auxiliary factors to achieve translation competence. Following nuclear export, pre-60S particles transiently rebind eIF6 at the inter-subunit interface, which blocks premature association with 40S subunits [240]. Removal of eIF6 involves the coordinated action of SBDS (Shwachman–Bodian–Diamond Syndrome)—a conserved AAA+ ATPase and EFL1 (a GTPase)—which induce conformational changes in the 60S subunit’s GTPase-associated center (GAC) [240]. Structural studies show SBDS binds near the sarcin-ricin loop (SRL) and the site where uL16/rpL10 docks onto the ribosome [241], destabilizing eIF6′s interactions with uL14/rpL23 [242] and eL24/rpL26 [240,243]. This displacement exposes the 60S subunit’s interface for 40S docking.

Concurrent with eIF6 release, rpL10/uL16 is assembled onto the 60S subunit in the cytoplasm and facilitates dissociation of the export adaptor NMD3 by competing for overlapping binding sites near the SRL. This step ensures NMD3′s nuclear export function is uncoupled from cytoplasmic ribosome activation [244]. The 60S subunit undergoes additional structural proofreading through Lso2, a conserved protein that transiently associates with the peptidyl transferase center (PTCe) to monitor rRNA folding [245,246]. Final maturation involves GTP hydrolysis by Efl1, which triggers the release of remaining assembly factors, like Tif6, and drives rotational adjustments in the 60S’s central protuberance, aligning it for proper 40S docking [223,240,247]. These structural reconfigurations are orchestrated by the ribosomal proteins uL5/rpL11 and rpL36A/eL42, which act as central stabilizers of the peptidyl transferase center (PTCe) and sarcin–ricin loop (SRL) during exchange of late maturation factors [240,248]. The integrity of uL5 is essential for proper recruitment of the SBDS and EFL1 (Elongation Factor-Like 1) remodeling factors, whereas depletion of eL42 blocks activation of the associated GTPase, preventing completion of the maturation cycle [240,248]. Through this coordinated action, rpL11/uL5 and rpL24/eL42 couple structural maturation of the large subunit to its acquisition of functional competence. Only after these checkpoints are satisfied can the 60S subunit participate in eIF5B-mediated joining with the 48S initiation complex, yielding a translationally active 80S ribosome [240].

Subunit joining and GTP hydrolysis by eIF5B promote the dissociation of remaining initiation factors, including eIF1A and eIF5B itself, yielding an 80S ribosome with initiator tRNA base-paired to the start codon in the P site and an empty A site ready to accept the first elongator aminoacyl-tRNA. At this point, the sequential series of initiation intermediates—activated mRNP, 43S PIC, scanning 48S complex, closed 48S at the start codon, and finally the assembled 80S ribosome—has been completed, and the ribosome transitions into the elongation phase of protein synthesis.

#### Non-Canonical Translation Initiation Mechanisms

These strategies bypass cap-dependent scanning, enabling translation under stress or viral infection. Here, we will refer to key mechanisms of non-canonical translation initiation. First, Internal Ribosome Entry Sites (IRESes) were first identified in poliovirus [249,250]. IRESes allow ribosomes to bind internally via structured RNA elements. Human IRESes in HIF1α and XIAP mRNAs sustain translation during hypoxia [251]. Second, Upstream Open Reading Frames (uORFs) regulate main ORF translation. In humans, ATF4 uORFs control stress responses, while yeast GCN4 uORFs modulate amino acid biosynthesis [140,252]. Third, although histone mRNA (H3 and H4) is cap-dependent to initiate translation, there is a unique structure in the 3′ stem-loop, which will be bound by the stem-loop binding protein (SLBP) to enhance translational activity. Also, human histone H2A employs an eIF4E-independent mechanism, while yeast lacks this pathway [140,252,253]. Fourth, non-AUG translation initiation broadens proteome diversity in both yeast and humans. In *Saccharomyces cerevisiae*, non-AUG start codons frequently generate N-terminally extended isoforms that acquire mitochondrial targeting signals (MTSs), producing a further pool of mitochondrial proteins and expanding the organellar proteome [254]. Genome-wide and proteomics analyses show that hundreds of yeast proteins can be translated from upstream near-cognate start codons, with the resulting extensions often responsible for mitochondrial targeting—a mechanism increasing the functional repertoire of mitochondrial components beyond canonical annotation [254]. In human cells, translation initiation from non-AUG codons, such as CUG, is well documented and can drive protein synthesis from transcripts bearing alternative start sites. For example, non-AUG initiation is involved in expressing oncogenes like MYC, which play crucial roles in cancer development and progression [255,256]. Ribosome profiling and reporter assays have revealed that non-AUG initiation contributes to the translation of diverse human proteins, particularly in stress conditions and malignancy, highlighting its regulatory and physiological importance [256].

### 6.2. Translation Elongation

**Figure 6:**
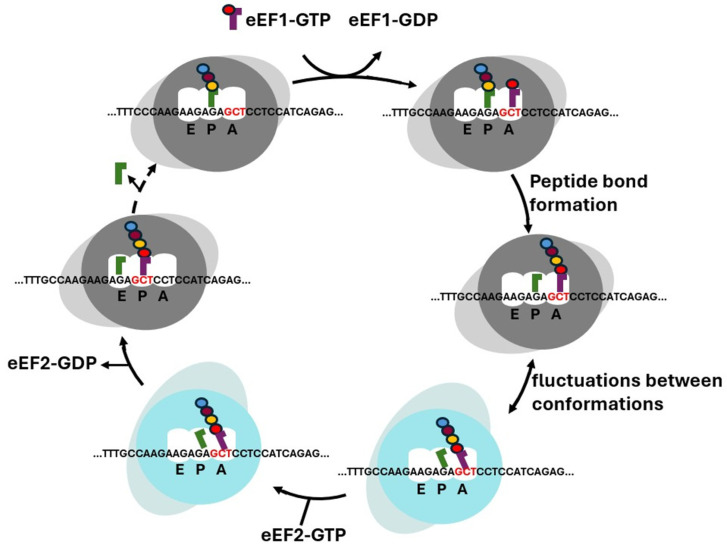
Conformational rearrangements of the eukaryotic ribosome during the elongation cycle drive processive movement along mRNA. The figure schematically depicts the eukaryotic translation elongation cycle, highlighting both peptide bond formation and the large-scale inter-subunit rotation that together propel the ribosome along the mRNA by one codon per cycle. Incoming aminoacyl-tRNA is first decoded in the A site of a non-rotated ribosome, where correct codon–anticodon pairing triggers peptide bond formation at the peptidyl transferase center. Peptide bond formation is followed by a major rearrangement in which the large and small ribosomal subunits undergo a substantial relative rotation (in blue) coupled to movement of A- and P-site tRNAs into hybrid states and re-positioning of the mRNA. In this rotated state, additional conformational changes—including an intra-subunit head swivel and factor-dependent transitions—prepare the ribosome for eEF2-driven translocation, during which GTP hydrolysis and the large inter-subunit rotation together mediate forward movement of the tRNAs and mRNA. After translocation, the ribosome returns to a non-rotated conformation with the peptidyl-tRNA now in the P site, completing one elongation cycle and positioning the next codon in the A site for subsequent decoding (modified from [260]).

Translation elongation in all kingdoms of life proceeds through a cyclic sequence of decoding, peptide bond formation, and ribosomal rearrangements (Figure 6). These basic steps not only ensure fidelity and efficiency of amino acid incorporation but also provide the molecular framework through which elongation rates can be tuned to meet varying translational demands [257,258,259]. Modulation of these steps allows cells to adjust translation kinetics in response to factors such as codon usage along an mRNA, properties of the emerging nascent chain, or changes in cellular state. Thus, the conserved structural transitions driving tRNA selection and catalysis are the same mechanisms exploited to fine-tune elongation speed and responsiveness, as revealed by ribosome profiling, quantitative proteomics, and live-cell imaging. Understanding how elongation dynamics can be adaptively modulated is central to the concept of RiboScreen^TM^ technology, and therefore, this section provides a more detailed discussion of elongation compared to other stages of the ribosome life cycle. Although the core principles of elongation are conserved between yeast and humans, species-specific adaptations in tRNA selection, ribosomal flexibility, and regulatory interactions illustrate evolutionary optimization of this regulatory capability.

The figure schematically depicts the eukaryotic translation elongation cycle, highlighting both peptide bond formation and the large-scale inter-subunit rotation that together propel the ribosome along the mRNA by one codon per cycle. Incoming aminoacyl-tRNA is first decoded in the A site of a non-rotated ribosome, where correct codon–anticodon pairing triggers peptide bond formation at the peptidyl transferase center. Peptide bond formation is followed by a major rearrangement in which the large and small ribosomal subunits undergo a substantial relative rotation (in blue) coupled to movement of A- and P-site tRNAs into hybrid states and re-positioning of the mRNA. In this rotated state, additional conformational changes—including an intra-subunit head swivel and factor-dependent transitions—prepare the ribosome for eEF2-driven translocation, during which GTP hydrolysis and the large inter-subunit rotation together mediate forward movement of the tRNAs and mRNA. After translocation, the ribosome returns to a non-rotated conformation with the peptidyl-tRNA now in the P site, completing one elongation cycle and positioning the next codon in the A site for subsequent decoding, modified from [260].

#### 6.2.1. Elongation Cycle Is Determined by Codon Recognition and tRNA Selection

During each elongation cycle, codon–anticodon recognition in the small subunit decoding center (A-site) sets the pace and fidelity of amino acid incorporation [257,258,259]. Eukaryotic elongation factor 1A (eEF1A), a GTPase, delivers aminoacyl-tRNAs (aa-tRNAs) and only correctly paired aa-RNAs trigger the conformational switch of the 40S head and shoulder from an open to a closed state, stabilizing the mRNA–tRNA mini-helix and accelerating GTP hydrolysis. Excellent reviews on correct tRNA selection and GTP hydrolysis are provided by several authors [261,262,263]. This induced-fit transition, together with a subsequent proofreading step, ensures that near-cognate tRNAs are rejected with high efficiency, keeping missense error rates in vivo near 10^−3^–10^−4^ per codon in yeast and mammalian cells [264,265]. The kinetics of this selection step at the A-site decoding site are strongly modulated by tRNA supply and modification. In budding yeast, codons decoded by abundant, properly modified tRNAs are recognized rapidly, whereas rare codons or hypomodified tRNAs prolong the initial binding and accommodation steps, increasing ribosome dwell time and producing local pauses along specific transcripts. Humans add an additional layer of regulation by deploying tissue-specific tRNA pools; for example, liver-enriched tRNAs accelerate decoding of metabolic mRNAs relative to other tissues, thereby shifting elongation rates in a gene- and organ-specific fashion without changing coding sequence [266,267,268]. These selection dynamics, governed by the efficiency of tRNA decoding and the match between codon usage and tRNA abundance, account for the up to 10- to 20-fold variation in effective elongation rates observed among yeast mRNAs. They also explain the more modest, yet measurable, influence of codon optimality—arising from differential tRNA supply and competition—on elongation speed in mammalian reporter systems [269,270,271].

#### 6.2.2. Peptide Bond Formation in the Peptidyl Transferase Center

Once a cognate aa-tRNA is fully accommodated, peptide bond formation proceeds in the peptidyl transferase center (PTCe) of the large subunit, a ribozyme built entirely from rRNA [271]. Structural and biochemical work supports a proton-wire mechanism in which conserved rRNA nucleotides guide proton transfers as the A-site amino acid α-amino group attacks the P-site ester bond formed by the previous cycle, with the overall rate limited primarily by accurate alignment of substrates rather than intrinsic chemical barriers [271,272]. The architecture of the PTCe is nearly superimposable between yeast and human ribosomes, with eukaryotic-specific expansion segments exerting subtle effects on substrate positioning that can fine-tune both catalytic efficiency and sensitivity to particular nascent sequences [31].

Certain amino acid contexts challenge this catalytic center and thereby slow elongation. Proline residues, especially in consecutive poly-Pro motifs, are poor peptide bond donors and acceptors, leading to prolonged PTCe dwell times and, in some cases, to discrete stalling events that require eIF5A, an elongation factor previously mislabeled as an initiation factor, to stabilize the transition state and re-accelerate bond formation [270,271]. Proteome-wide analyses in yeast show that mRNAs encoding proline-rich or otherwise “difficult” motifs exhibit lower effective elongation rates for a given ribosome density, illustrating how PTCe-encoded chemistry contributes to the spread of elongation speeds that emerge along the mRNA. Perturbations of key PTCe nucleotides in yeast 25S rRNA similarly reduce peptidyl-transfer rates and trigger quality-control pathways, linking core catalytic efficiency to the surveillance systems that respond to pathological stalls [271,273].

#### 6.2.3. Nascent Peptide Transit Through the Exit Tunnel

As the polypeptide grows, it traverses the ribosomal exit tunnel, an 80–100 Å conduit whose geometry and electrostatics strongly influence local elongation kinetics. In yeast, the tunnel narrows to about 10 Å at a constriction formed by rRNA and ribosomal protein loops and widens to ~20 Å near the exit, accommodating approximately 30–40 residues in an extended conformation, while eukaryote-specific expansions such as the uL4 loop create a second constriction that modulates how secondary structure emerges [274,275]. Human ribosomes incorporate distinct tunnel-lining proteins (for example, eL39 in place of the bacterial uL23 loop), yielding a slightly narrower path that is particularly attuned to the passage of bulky or strongly charged segments typical of secretory and membrane proteins [275].

Interactions between the nascent chain and the tunnel walls are key determinants of elongation rate heterogeneity [276]. Positively charged segments, such as poly-lysine stretches or arginine-rich domains, engage the negatively charged rRNA backbone and basic side chains of tunnel proteins, slowing translocation and increasing local ribosome dwell time, whereas acidic segments tend to transit more rapidly [173,277]. Genome-scale analysis in yeast shows that proteins with higher overall isoelectric points—most prominently ribosomal proteins—are synthesized more slowly per bound ribosome than negatively charged proteins, despite similar or higher tRNA adaptation indices, implying that exit-tunnel friction is as important a determinant of elongation speed as codon usage [274]. In human cells, signal peptides and transmembrane segments that contact the tunnel contribute to programmed pauses that coordinate recruitment of signal recognition particle or translacon components, embedding targeted slowdowns in elongation to synchronize translation with targeting and folding [274].

#### 6.2.4. Ribosomal Subunit Dynamics and Translocation

Coupling between chemistry and motion is mediated by large-scale rearrangements of ribosomal subunits during each elongation cycle. This phenomenon has been explored initially in bacterial systems in great detail by the Niehaus group and Subramaniam group [278,279,280], and in the last decade, our knowledge of eukaryotic subunit dynamics has been greatly enhanced. After peptide bond formation, the eukaryotic ribosome enters a rotated, hybrid-tRNA state in which the 40S subunit ratchets by roughly 8–9° relative to the 60S [269,281], and the 40S head undergoes a characteristic swivel that positions tRNAs in A/P and P/E sites [269,282]. Cryo-EM reconstructions in yeast and human systems reveal that these conformational transitions are guided by conserved rRNA bridges between subunits, and the exact rotation axes and interaction networks are modulated by eukaryote-specific rRNA expansion segments and ribosomal proteins, which can tune the dwell time in individual states [195,283,284,285].

eEF2 binds preferentially to the rotated ribosome and, upon GTP hydrolysis, drives reverse rotation and head back-swiveling, completing translocation and restoring classical P/P and E/E tRNA positions with an empty A-site ready for the next decoding event [281,286,287]. Phosphorylation of eEF2-by-eEF2 kinase under nutrient deprivation or stress increases the lifetime of complexes in rotated or pre-translocation states, globally slowing elongation and enhancing the likelihood of ribosome collisions at intrinsically slow motifs [270,288,289]. Single-molecule studies further indicate that yeast ribosomes spontaneously fluctuate between rotated and unrotated states even in the absence of elongation factors, with bound tRNAs stabilizing subsets of these conformations; such fluctuations set the baseline mechanical timescale on which codon-dependent decoding and nascent-chain effects act to generate site-specific pauses [290]. Differences in the tightness and timing of these motions between yeast and humans likely contribute to species-specific propensities for frameshifting and may buffer certain human transcripts against extreme local slowdowns despite challenging sequence features [283].

#### 6.2.5. Natural Translational Errors: Missense and Readthrough

Elongation fidelity arises from the combined contributions of decoding accuracy, proofreading, and quality-control pathways that monitor stalled or collided ribosomes [291,292]. In this context, *fidelity* refers to the precision with which the ribosome incorporates the correct amino acid corresponding to each codon in the mRNA, ensuring accurate protein synthesis.

In yeast, reporter-based and mass-spectrometry-based analyses show that near-cognate tRNAs are occasionally accepted at the A-site, yielding codon-specific missense error frequencies typically in the 10^−7^–10^−5^ range per codon, with higher rates for a small subset of error-prone codons. These error spectra depend strongly on codon identity, local sequence context, and the relative abundances of cognate and near-cognate tRNAs [264,293]. Comparable analyses in mammalian cells, integrating ribosome profiling with proteome-wide misincorporation measurements, support a similar average error scale, although local rates can rise at problematic codons, under amino acid limitation, or during stress when tRNA charging and proofreading are perturbed [273,276]. A more detailed discussion of readthrough of stop codons and premature stop codons/nonsense codons will be presented below in the section on the termination of protein synthesis.

#### 6.2.6. Methods to Quantify Elongation Rates

A change in elongation rates for an individual mRNA species for decades has been disputed. However, a diverse toolkit now exists to quantify elongation kinetics from single-molecule to organismal scales, each still associated with specific strengths and biases [273,276]. In vitro, translation-competent mammalian lysates and luciferase reporters have been used to estimate elongation speeds by dividing open reading frame (ORF) length by the delay to detectable activity, yielding apparent rates around 0.7 amino acids per second in HeLa extracts and 1.5 amino acids per second in rabbit reticulocyte lysate, although these values likely underestimate in vivo speeds because they conflate initiation, elongation, and folding [273,294]. Heavy-isotope labeling in lysates or SILAC-type approaches in cells enable more global rate estimates, but again integrate over multiple steps and often suffer from modest temporal resolution [173].

Ribosome profiling combined with pharmacological separation of initiation and elongation allows transcriptome-wide inference of elongation rates in vivo. In mouse embryonic stem cells, harringtonine run-off followed by footprint sequencing gave average elongation rates of about 5–6 amino acids per second [295], whereas in yeast, pSILAC-calibrated ribosome profiling in exponentially growing cells yielded effective elongation rates around 2.5 amino acids per second and revealed up to 20-fold variation between transcripts [173]. Methodological details matter, and that is exemplified by the observation that cycloheximide, often used to “freeze” ribosomes, can introduce codon-dependent biases, prompting the adoption of flash freezing or alternative arrest strategies to improve the accuracy of analysis [273,296].

Live-cell single-molecule imaging of translation provides direct kinematic readouts at the level of individual mRNAs. SINAPs reporters, which label nascent peptide arrays together with their mRNAs, have been used to estimate elongation rates by measuring ribosome run-off after initiation block, yielding values of roughly 4–5 amino acids per second for mammalian reporters under conditions where single ribosomes translate isolated ORFs [273]. An advanced ‘stopless ORF’ circular RNA system that traps ribosomes on a continuous coding loop has made it possible to track individual ribosomes over many thousands of codons, revealing single-ribosome elongation rates in a similar range and uncovering ribosome cooperativity, whereby transient collisions between multiple ribosomes on difficult-to-translate regions reduce pausing and promote efficient translation [273,288,289].

At the organismal level, systemic administration of harringtonine followed by tissue-specific ribosome profiling in mice shows that elongation rates differ by organ, with liver, kidney, and skeletal muscle exhibiting average rates of approximately 6.8, 5.0, and 4.3 amino acids per second, respectively, and with liver elongation slowing by about 20% between young and aged animals. These measurements indicate that average elongation rates are organ- and age-dependent, and, together with other ribosome profiling studies, support the view that elongation is also transcript-specific [273,297].

#### 6.2.7. Determinants of Elongation Rates and Magnitude of Variation

When these mechanistic modules are viewed together, differential elongation rates emerge naturally from how codon recognition, PTCe catalysis, tunnel transit, and subunit dynamics are wired into a single cycle rather than from any one factor alone.

Combining protein synthesis rates with ribosome occupancies in eukaryotic cells allows direct inference of initiation and elongation contributions for individual ORFs. In budding yeast, the per-mRNA initiation rate spans roughly two orders of magnitude, while the effective elongation rate has been inferred to vary by up to about twenty-fold across transcripts, and similar transcript-to-transcript differences in elongation speed are suggested in human cells. However, these estimates come largely from bulk ribosome-profiling–based approaches and reporter constructs, which may not fully capture the true distribution of elongation rates for endogenous mRNAs in vivo [173,298]. Ribosome-profiling, modeling, and single-molecule imaging show that elongation is highly non-uniform along a single mRNA, and that codon usage, tRNA pools, amino acid sequence, mRNA structure, and regulated pausing collectively tune the effective elongation rate on that transcript, which, in many experimentally tested cases, can increase or decrease the amount of protein produced from the same mRNA [99,296]. Under baseline conditions, the protein synthesis rate nonetheless increases nearly linearly with ribosome density across most examined yeast transcripts, indicating that ribosome collisions are usually rare in this regime and that variation in elongation speed primarily tunes protein flux at a given ribosome allocation [173].

Codon usage and tRNA adaptation indices (tAI) positively correlate with elongation speed, consistent with faster decoding at codons matched to abundant tRNAs. In yeast, these codon-centric metrics explain a substantial fraction of the variance in elongation rates, and ribosome profiling confirms longer dwell times on non-optimal codons [299,300]. In mammalian cells, however, the impact of codon optimality on instantaneous elongation appears more modest: circular RNA reporters with synonymous ORFs show only about 6–16% slower elongation on non-optimal sequences, yet codon optimization can markedly increase protein production via enhanced mRNA stability and initiation, highlighting species-specific deployment of elongation-linked regulation [173].

Properties of the nascent polypeptide make contributions comparable in magnitude to codon and tRNA metrics. In yeast, the isoelectric point of the emerging protein is strongly anti-correlated with the elongation-rate surrogate (protein synthesis rate divided by ribosome density): negatively charged proteins are synthesized up to roughly two-fold faster than positively charged ones at a given ribosome density. Ribosomal protein (RP) mRNAs illustrate this principle: they have very high tRNA adaptation indices, yet exhibit relatively slow elongation and lower protein output than non-RP transcripts with similar ribosome densities, consistent with positively charged nascent chains experiencing enhanced friction in the negatively charged exit tunnel. Amino acid side-chain size also matters: small, nonpolar residues such as alanine and glycine are associated with higher elongation speeds, whereas bulky hydrophobic residues and proline correlate with slower progression [173,301].

mRNA secondary structure exerts a nuanced influence. Globally, transcripts with more predicted secondary structure along their ORFs tend to have higher elongation-rate surrogates, suggesting that structured mRNAs may help maintain favorable ribosome flux and prevent traffic jams. These observations imply that cells exploit both codon choice and mRNA structure to sculpt spatially heterogeneous elongation landscapes [173].

Physiological and environmental conditions add additional layers of control. In yeast, pSILAC-calibrated TASEP modeling and ribosome profiling indicate that deletion of ribosomal protein genes shifts initiation and elongation parameters in gene-specific ways [173,302,303]. In addition, in strains with globally increased or decreased translation, there is little evidence that ribosome collisions broadly curtail protein synthesis; instead, transcripts appear to be evolutionarily tuned to operate below collision-dominated regimes. On the other hand, in mammals, nutrient deprivation, amino acid limitation, and stress-activated kinases such as GCN2 or eEF2 kinase reduce elongation through tRNA deacylation or eEF2 phosphorylation, leading to slower ribosomes, increased collision frequency at difficult motifs, and engagement of ribosome-associated quality-control and decay pathways [149,173,304].

Across these determinants, the net dynamic range of elongation rates is substantial. In yeast, effective elongation rates inferred from combined pSILAC and ribosome profiling span roughly a 20-fold range, from slow transcripts enriched for positive charge and non-optimal codons to fast, highly optimized metabolic enzymes. In mammalian tissues, organ-level averages range from about 4 to nearly 7 amino acids per second, with within-tissue variation further stratified by codon usage, RNA structure, nascent peptide composition, and local regulatory factors [173,273,297,305].

#### 6.2.8. Differential Elongation as a Regulatory Layer

The integration of ribosome profiling, quantitative proteomics, and live-cell imaging has reframed elongation as an active regulatory axis that intersects with initiation, mRNA stability, and protein quality control. In yeast, synonymous fluorescent reporters designed with a neural-network model of ribosome dwell times demonstrate that codon-encoded elongation speed alone can create up to a 12-fold range in protein output from transcripts sharing identical promoters and 5′ UTRs. For these reporters, differences in mRNA abundance due to codon-optimality-mediated decay account for only part of the effect; translation efficiency, defined as proteins produced per mRNA per time, still varies by approximately six-fold across codon variants, indicating that slow elongation directly restricts protein output [301].

Polysome profiling of these synonymous reporters reveals that mRNAs with slower codons are associated with fewer ribosomes than predicted by simple exclusion-process models, ruling out passive traffic jams as the main mechanism and implicating feedback that limits initiation on slow-elongating transcripts. Deletion of canonical collision-sensing quality-control factors (such as Hel2, Mbf1, and Gcn1) has little effect on the differential output of fast versus slow reporters, whereas a genome-wide CRISPRi screen identifies multiple translation initiation factors, including eIF3 subunits, whose partial depletion selectively reduces the disadvantage of slow codons. Introducing 5′-UTR stem-loop structures that weaken initiation similarly attenuates the impact of codon-encoded elongation speed, revealing a parameter regime in which protein output is predominantly initiation-limited and a complementary regime in which elongation is rate-limiting [301,306].

Mapping endogenous yeast genes into this initiation–elongation space by combining UTR-driven reporter measurements with codon-based elongation predictions and absolute protein synthesis rates shows that a substantial fraction of genes operate in a zone where elongation constrains translation efficiency [173,301]. For transcripts with above-median elongation speeds, translation efficiency correlates more strongly with UTR-determined initiation strength, whereas for those with slower-than-median elongation, changes in initiation strength have diminished impact, consistent with an elongation-limited regime. This arrangement suggests co-evolution of UTRs and coding sequences such that highly initiated transcripts often encode sequences with relatively fast elongation, whereas transcripts with intrinsically slow elongation are buffered against further increases in initiation rate [301].

In mammalian systems, analogous coupling of elongation dynamics with stress signaling and mRNA decay is well documented. ZNF598, a central E3 ubiquitin ligase that senses collided ribosomes, recognizes stalls and collisions that arise on difficult-to-translate sequences such as poly-lysine tracts, poly-A stretches, and, in many contexts, pathogenic repeat-encoded dipeptides like poly-GR (glycine-arginine) and poly-PR (proline-arginine). These events trigger ribosome-associated quality control: ZNF598-dependent ubiquitination of small-subunit ribosomal proteins marks stalled complexes for downstream processing, which can include nascent chain degradation and endonucleolytic mRNA cleavage followed by decay through no-go/non-stop-like pathways. In parallel, quality-control factors such as GIGYF2–4EHP repress further initiation on problematic mRNAs, and broader stress-responsive kinases such as GCN2 can inhibit global initiation via eIF2α phosphorylation, providing feedback from elongation stress to initiation control. Conversely, regulated stalls in upstream open reading frames—including those created by polyproline or other hard-to-translate motifs—can modulate re-initiation at downstream main ORFs. Depending on uORF configuration and cellular conditions (for example, nutrient or polyamine availability), such programmed pausing can either dampen or enhance main-ORF translation, allowing cells to convert metabolic and stress cues into gene-specific translational outputs via controlled modulation of elongation and re-initiation [273,307].

Together, these observations position differential elongation rates as a bona fide regulatory layer that complements initiation and mRNA turnover in shaping gene expression programs. By modulating codon usage, tRNA supply, RNA structure, and nascent peptide features, cells can, in many experimentally documented cases, retune elongation kinetics on selected transcripts to alter protein production without changing mRNA levels. Global perturbations of initiation, as in stress responses or growth-rate transitions, are predicted and in several systems observed to propagate through this landscape in a non-uniform fashion, differentially affecting transcripts depending on their initiation strength and codon/elongation properties—for example, by preferentially revealing elongation bottlenecks on specific stress-sensitive mRNAs [301,308,309]. This interplay between initiation and elongation, together with the associated quality-control reactions at stalled or collided ribosomes, provides the conceptual framework within which RiboScreen^TM^ technology interrogates and perturbs translation elongation as a dynamic, regulatable determinant of protein production from a given mRNA or protein of interest (POI).

### 6.3. Translation Termination and Recycling

**Figure 7:**
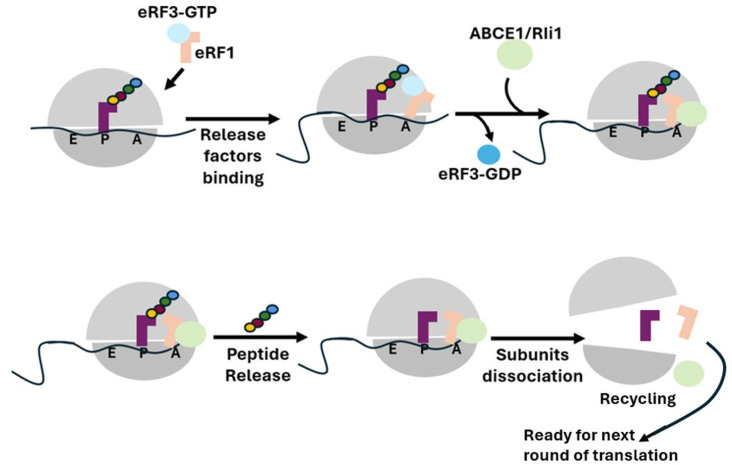
Translation termination in eukaryotes: eRF1–eRF3–ABCE1–driven stop codon recognition, peptide release, and ribosome recycling. The figure illustrates the coupled stages of translation termination and ribosome recycling in eukaryotes, emphasizing how eRF1 and eRF3 promote accurate stop codon recognition to prevent readthrough. When a termination codon enters the A site of a pre-termination complex bearing a peptidyl-tRNA in the P site, the eRF1–eRF3–GTP ternary complex is recruited to the empty A site, where eRF3 greatly enhances the affinity and selectivity of eRF1 for stop codons over competing near-cognate aminoacyl-tRNAs. GTP hydrolysis on eRF3 is stimulated on the ribosome, leading to eRF3·GDP dissociation and allowing a rearrangement that positions the catalytic GGQ motif of eRF1 into the peptidyl transferase center to trigger rapid and efficient peptide release, thereby minimizing stop-codon readthrough. Binding of the ATP-binding cassette protein ABCE1/Rli1 to the post-termination complex further accelerates eRF1-mediated peptide release and then, upon ATP hydrolysis, drives dissociation of the large and small subunit, which are recycled, with initiation factors re-binding to the 40S subunit to support a new round of translation on the same or a different mRNA (modified from 232).

Translation termination is a critical phase of protein synthesis, ensuring precise release of nascent polypeptides and recycling of ribosomal subunits. While the core machinery is conserved between yeast and humans, species-specific adaptations in termination factors, regulatory networks, and quality control mechanisms highlight evolutionary tuning of this process. Recent advances in structural biology and ribosome profiling have illuminated how termination fidelity is maintained, how premature termination codons (PTCs) are managed, and how ribosomes are recycled for subsequent rounds of translation (Figure 7) [233,235,310,311].

#### 6.3.1. Termination Factors and Stop Codon Recognition

In eukaryotes, termination is mediated by eukaryotic release factor 1 (eRF1) and its GTPase partner eRF3. eRF1 recognizes all three stop codons (UAA, UAG, UGA) through a conserved tripeptide motif (TASNIKS) in its N-terminal domain, while its GGQ motif catalyzes peptidyl-tRNA hydrolysis [312,313,314]. eRF3 binds GTP and stabilizes eRF1 on the ribosome, enhancing termination efficiency by 10- to 50-fold. Structural studies reveal that human eRF1 adopts a tRNA-like conformation upon binding eRF3, enabling precise positioning in the ribosomal A-site.

Yeast eRF1 (Sup45) shares 65% sequence identity with human eRF1, but exhibits weaker discrimination against near-cognate tRNAs, contributing to higher basal readthrough rates at cognate stop codons ~0.1–1% in yeast vs. ~0.01% in humans [315,316,317,318].

#### 6.3.2. Termination Fidelity at Cognate Stop Codons

Termination in eukaryotic translation is highly accurate, ensuring that most ribosomes correctly recognize stop codons and release completed polypeptides. In yeast, genome-wide ribosome profiling reveals that only a minor fraction of ribosomes extend beyond natural termination sites, with average basal readthrough frequencies per stop codon typically below 0.1%, and exceptional cases reaching ~1%. These estimates are consistent with classical reporter assays. Human cells similarly display high termination efficiency, with basal readthrough at natural stops usually below 0.01–0.1% and modest increases (up to ~0.5–1%) seen only at permissive sequence contexts [319,320,321,322].

Termination efficiency and readthrough propensity are strongly influenced by the local sequence environment. Nucleotides immediately downstream of the stop codon—particularly positions +4 to +9—modulate how tightly eukaryotic release factors (eRF1 and eRF3) interact with the ribosome. Purine residues at +4 tend to strengthen termination, while certain U- or C-rich sequences can promote low-level readthrough [323,324]. Cryo-EM data indicate that, in humans, eRF1 forms stable interactions within the 40S decoding center that minimize near-cognate tRNA misincorporation, yielding termination fidelities exceeding 99.9%. Yeast ribosomes achieve somewhat faster peptide release kinetics (half-time ~50 ms) than humans (~200 ms), reflecting subtle differences in eRF3-GTPase activation [318].

Although rare, endogenous readthrough at cognate stop codons can serve regulated biological functions. In yeast, approximately 0.3% of mRNAs undergo programmed stop codon readthrough to generate C-terminally extended isoforms, for example PDE2, which acquires a peroxisomal targeting signal. In human cells, stress conditions such as hypoxia enhance readthrough at specific sites (e.g., the UGA stop codon of *HIF1α*), producing functionally distinct protein variants [325]. The [PSI^+^] prion in yeast demonstrates that release-factor sequestration (Sup35/eRF3) can globally reduce termination fidelity, revealing that this process is tunable [326].

#### 6.3.3. Termination at Premature Stop Codons and NMD

Premature termination codons (PTCs), arising from nonsense mutations or splicing errors, are decoded with intrinsic accuracy comparable to that of natural stops. However, their cellular fates differ profoundly. Transcripts harboring PTCs are predominantly recognized and degraded by the nonsense-mediated mRNA decay (NMD) pathway, which limits the accumulation of truncated, potentially deleterious proteins [327,328,329].

The decision between continued translation at a PTC or mRNA elimination via NMD depends on both the local termination context and, particularly in humans, the position of the premature stop codon relative to exon–exon junction complexes (EJCs). In human cells, PTCs located more than ~50–55 nt upstream of the final exon–exon junction efficiently trigger NMD [327,330]. Yeast and human systems use homologous but mechanistically distinct NMD factors: in yeast, the UPF1–3 complex promotes termination fidelity and eRF1 release, whereas in mammals, UPF3B can destabilize eRF1–eRF3 interactions, favoring transcript degradation. ATP hydrolysis by UPF1 is essential for NMD in yeast but not in human cells, indicating evolutionary divergence of regulatory control [330].

PTCs residing in suboptimal sequence environments—such as those with U- or C-rich nucleotides immediately downstream—show a reduction in termination efficiency to about 70–90%, and consequently a small increase in basal readthrough (typically 0.1–1%, occasionally higher under drug treatment). Pharmacological compounds like aminoglycosides or ataluren can further enhance near-cognate tRNA incorporation, restoring a fraction of full-length protein [322,331]. Nonetheless, for most PTC-containing mRNAs, NMD-mediated clearance dominates over translational readthrough, resulting in very low levels of protein rescue under untreated conditions [320].

#### 6.3.4. Ribosome Recycling After Termination

Post-termination ribosomes must release mRNA and tRNA and dissociate into subunits. In yeast, recycling is initiated by initiation factors eIF3, eIF1, and eIF1A, which bind the 40S subunit and displace deacylated tRNA. eIF3′s N-terminal domain acts as a wedge to split subunits, a process accelerated by ABCE1/Rli1 in an ATP-dependent manner [332,333]. Humans rely more heavily on ABCE1, which couples GTP hydrolysis by eRF3 to dissociate subunits. Structural studies show that ABCE1′s iron-sulfur domain docks onto the ribosomal intersubunit bridge, leveraging ATP hydrolysis to destabilize assembled ribosomes [334,335].

Recycling of ribosomes loaded onto PTC mRNAs differs: yeast employs Dom34/Hbs1 to dissociate stalled complexes, while humans use Pelota/HBS1L [336]. Both systems also require ABCE1/Rli1, but human Pelota shows stronger dependence on GTPase activity. Intriguingly, yeast eIF3 can recycle post-termination ribosomes without ABCE1 under low-stress conditions, whereas human ribosomes strictly require ABCE1 even with optimal activity of the initiation factors involved [333,336].

## 7. Ribosome Specialization

We have discussed ribosomes as universally present core protein-synthesizing factories of all living cells, and as such, they have long captivated molecular biologists not just for their essential role in protein production, but also for the astonishing scale at which they operate. In the model yeast *Saccharomyces cerevisiae*, the number of ribosomes per cell has been quantified by several groups through diverse cell-cycle stages and growth regimes, with consensus counts ranging typically between 150,000 and 350,000, and converging on values around 200.000 in vigorously dividing haploid strains [103,177,337,338]. We have also learned that these organelles are by far the most plentiful machines composed by elaborate transcriptional and translational control of their RNA and protein components, with their numbers subject to dynamic regulatory programs that reflect growth conditions, metabolic state, and the cell’s position in the cell cycle [177]. In terms of sheer abundance, human cells exhibit even greater numbers. For example, studies of rapidly proliferating Jurkat T lymphocytes estimate that each cell harbors on the order of 2 million ribosomes [339], and some authors report even higher numbers, up to 10.000.000 ribosomes per cell [340,341]. Clearly, these numbers are not merely of academic interest—they underpin the enormous energy and resource allocation that cells devote to gene expression at the level of translation [177,342].

Given these staggering numbers and the ribosome’s essential function in gene expression, early in the history of ribosome biology, it was asked whether all these ribosomes were functionally and structurally identical or whether meaningful diversity might exist within the ribosome population itself. The intellectual roots of this question reach back to Francis Crick’s bold “one gene–one ribosome–one protein” hypothesis [343], intended to explain early findings from protein synthesis. While this early model was swiftly disproved—seminal experiments demonstrated unambiguously that ribosomes are not fixed to synthesize just a single protein, but instead exhibit versatile mRNA decoding—Crick’s intuition opened the avenue for considering ribosome diversity as a possible regulatory mechanism in gene expression. In the decades since, and especially as the analytical and structural tools of molecular biology have become increasingly powerful, the field has amassed persuasive evidence that ribosomes do not merely form a monolithic pool in the cytoplasm; rather, ribosome heterogeneity is a widespread phenomenon whose biological consequences are just beginning to be understood [104,105,110,344,345,346].

Ribosomal heterogeneity arises at multiple, interrelated molecular levels. In lower and higher eukaryotes, plants in particular, many ribosomal proteins are encoded by gene families with paralogous members. These paralogs are often differentially expressed depending on the cell or tissue type, developmental stage, or in response to environmental conditions, permitting the cell to assemble variant ribosomes equipped, possibly with subtly distinct functional capacities [347,348]. Recent technological progress in high-throughput proteomics has allowed the in-depth investigation of these paralogs, confirming that the spectrum of ribosomal protein isoforms is far richer than previously believed, and that specific paralog compositions often correlate with the functional specialization of particular tissues or developmental programs [116,349]. In parallel, the ribosomal RNA (rRNA) components themselves can exhibit diversity, arising through alternative rDNA gene copies, developmental or stress-dependent alternative splicing, or chemical modifications—in this review, we have already discussed the most notable ones, 2′-O-methylation and pseudouridylation. Variation in rRNA modification was revealed by a combination of classical enzymatic mapping and, more recently, direct RNA sequencing approaches [345,350,351,352].

It now becomes increasingly clear that ribosomal diversity does not arise randomly, but rather, results from regulated modifications and gene expression programs that respond to physiological and pathological cues. The actual mechanisms producing this diversity include not just the differential expression of RP paralogs and rRNA isoforms, but also extensive post-translational modification of ribosomal proteins, such as phosphorylation, acetylation, and ubiquitination [353,354,355]. In fact, in all domains of life, a single ribosomal protein can harbor as many as 15 different modification sites [356,357]. It has been suggested that these molecular variants modulate ribosome assembly, activity, and may even, during translation, contribute to the processing of particular mRNA structures or sequence elements [358]. Furthermore, the final ribosomal identity can be influenced by the association of the ribosome with auxiliary translational factors or regulatory proteins, which may selectively occupy certain ribosome sites depending on cellular state, thus modulating the translation process in ways that can be highly context specific [344,359].

The presence and scope of ribosome heterogeneity were historically hinted at in studies of tissue-specific expression and developmentally regulated ribosomal components. However, only with the surge of “ribosome omics” technologies have the full spectrum and scale of heterogeneity been illuminated. An outstanding demonstration of this is provided by the Wang laboratory, which developed and applied the “RibosomeR” proteomics approach for the systematic analysis of ribosomal protein complexes in vivo [347]. By analyzing ribosomes purified from fat, spleen, heart, and muscle tissues, these researchers discovered robust and functionally significant differences in ribosomal protein composition between tissues; these differences, in turn, conferred distinct translational profiles likely to meet the particular functional requirements of different cell lineages [347].

The array of experimental tools and techniques developed to investigate ribosome heterogeneity has blossomed significantly over the past decade, with each method offering unique kinds of insight. Quantitative mass spectrometry-based proteomics can measure not only the presence or absence of core proteins, but also resolve variations in the abundance of key isoforms or post-translationally modified species, even in relatively rare ribosome subpopulations [349,360,361,362,363]. Ribosome profiling, better known as Ribo-seq, revolutionized translational studies by enabling researchers to “map” translation at nucleotide resolution across the entire transcriptome. By combining Ribo-seq with immunoprecipitation using subunit- or paralog-specific antibodies, it has become possible to pinpoint which subsets of mRNAs are preferentially translated by which subpopulations of ribosomes, illuminating potential mechanisms for selective translational control [364,365,366].

Within structural biology, the method of cryo-electron microscopy (cryo-EM) has undergone a renaissance and has now achieved near-atomic resolution of individual ribosomes. This enables the visualization of subtle structural differences that correspond to specific protein variants or rRNA modification states [359,367]. Cryo-EM has thus made possible the head-to-head comparison of ribosomes across distinct cellular conditions, including those from healthy versus disease tissues [368]. Complementary physical and biochemical techniques, such as single-molecule FRET (smFRET) and atomic force microscopy (AFM), allow researchers to probe dynamic conformational states and functional kinetics of individual ribosome molecules, further mapping the relationship between structural diversity and translation dynamics in real-time. Similarly, the growth of direct and long-read RNA sequencing—particularly with nanopore technologies—has been instrumental in distinguishing generation of rRNA splice forms as well as mapping the precise landscape of rRNA modifications that mark specialized ribosome subtypes [359,369,370,371,372].

Critical to the growing field of ribosome diversity is the ability to selectively isolate and enrich specific ribosome subclasses from complex mixtures—a feat accomplished by affinity-based biochemical purification methods such as RAPIDASH, RAPPL, and TREX, which use antibodies or unique sequence tags to “pull down” ribosomes carrying specific protein or RNA signatures [359,373,374,375]. This capacity to fractionate ribosome populations before downstream analysis has greatly improved our ability to parse and functionally interrogate small but critical subpopulations that might otherwise go undetected in pooled ribosome samples.

While the presence of biochemical and structural heterogeneity among ribosomes is now acknowledged, the more profound insight is that this diversity may in many cases translate directly into functional specialization—giving rise to what are now termed “specialized ribosomes.” In this context, “specialization” implies that compositionally distinct ribosomes, be it through unique ribosomal protein variant combination, differences in rRNA sequence or modification, or preferential association with certain cofactors, exhibit distinct translational profiles due to their altered ability to recognize, bind, initiate or elongate selected mRNA transcript classes [104,358,359,364].

Some of the clearest experimental evidence in support of this paradigm comes from studies of rRNA methylation and developmental regulation of ribosomal protein content. For example, one particularly well-studied instance is the selective methylation of a single nucleotide residue in the 18S rRNA in mammalian ribosomes, which has been shown to modulate translational preference between distinct mRNA subsets, thus fine-tuning the proteome output in response to environmental cues [376]. Another landmark finding involves the tissue-specific incorporation of the ribosomal protein RPL38, which is required for the translation of a critical subset of Hox mRNAs governing vertebrate axis patterning; knockouts of RPL38 disrupt Hox translation without a general block to protein synthesis, providing a classical proof of principle for ribosome-driven selectivity in mRNA translation [104,377]. Tissue survey studies have further demonstrated that the unique proteomic signature of variant ribosomes in specialized tissues directs translation toward cell- and organ-specific proteins crucial for physiology and adaptation [347,349].

Synthetic biology and bacterial molecular engineering have capitalized on this concept by constructing orthogonal ribosome-mRNA pairs, where engineered ribosomes selectively translate only synthetic mRNAs with matching signals, independently from the cell’s endogenous translation machinery. These orthogonal systems have, in effect, created an entirely novel translation program within living cells, experimentally validating the idea that engineered ribosome composition can dictate translation specificity [371,378,379,380].

Specialized ribosomes are further implicated in the context of disease, as the long-standing observation that inherited mutations in ribosomal protein genes cause rare disorders called ribosomopathies, such as Diamond–Blackfan anemia, laid the groundwork for appreciating the significance of ribosome specialization in human disease [341,381,382]. More recent studies have extended this realization into cancer biology, where “oncoribosomes”, distinguished by their somatic mutations in ribosomal proteins or altered rRNA modification profiles, are now recognized as a hallmark of certain cancers and may actively promote tumorigenesis by reshaping the cellular translational program in favor of proliferation, survival, or metastasis [6,135,383].

Key mechanistic studies have uncovered how somatic mutations in genes encoding ribosomal proteins—such as RPL10-R98S in pediatric T-cell acute lymphoblastic leukemia and recurrent mutations in RPL5 or RPL11 in various carcinomas—selectively shift the translational output toward pro-oncogenic or stress-adaptive mRNAs, conferring growth or survival advantage [384,381,6.] Similarly, alterations in rRNA modification patterns or rDNA gene dosage can modulate ribosomal specificity and fidelity in ways that support cancer cell adaptability [135,347]. Analyses employing advanced mass spectrometry and proteomics have revealed that cancer tissues often display a distinctive “ribosomal signature” distinct from the adjacent normal tissue, and these onco-ribosomal populations often correlate with distinct translational programs associated with malignancy [384,385]. This growing body of evidence supports the view that onco-ribosomes are a disease-associated form of specialized ribosomes and may even serve as drivers for specific cancer phenotypes, providing an entirely novel set of potential biomarkers or therapeutic targets, especially for precision oncology [386,387].

The concept that ribosomes themselves encode regulatory information—a “ribosome code”—thus constitutes a disruptive and transformative shift in our view of genetic information flow, compelling a re-examination of the classic central dogma of molecular biology. The presence and mechanistic importance of ribosomal specialization add a newly appreciated, orthogonal layer of regulation atop DNA and transcriptome-level control, suggesting that cells can dynamically tune their translational machinery for adaptive gene expression at the level of the ribosome itself [104,110,347,358,359].

In terms of basic science, this “ribosome code” model opens up new conceptual terrain—for instance, by establishing that the cellular proteome can be partly defined by the repertoire of ribosomes in use, rather than strictly by the abundance or structure of transcripts present. This in turn reconfigures how we think about cellular adaptation, developmental programming, and the evolution of regulatory complexity. The integration of transcriptomics, ribosome profiling, and advanced proteomics across entire systems is beginning to illuminate the modularity and specificity governing gene expression at the level of translation, suggesting that translational control may play a far larger role in cell identity and plasticity than previously suspected [347,359]. Yet, for all this progress, major questions remain open. The precise molecular mechanisms by which ribosome specialization brings about mRNA selectivity, the contextual determinants of subpopulation assembly, and the full downstream phenotypic impact of ribosome diversity are only now being unraveled, requiring ever more sophisticated and integrated experimental approaches. In particular, it remains to be established whether a given mRNA associated with a variant rather than a standard ribosome forms this one ribosome that preferentially translates this one protein of which Francis Crick speculated on [343]. With the insight only given to a genius, Crick anticipated a model in which a specific mRNA species not only serves as a translational template but also contributes to the structural and functional tuning of the ribosome itself. Upon ribosome engagement, the mRNA can influence local ribosomal dynamics—through its secondary structure, codon composition, or interaction with rRNA expansion segments and ribosomal proteins—thereby promoting a ribosome state optimized for its own efficient translation. Within the cellular pool, ribosomes generally possess the intrinsic capability to translate any mRNA; however, subtle variations in ribosomal protein composition, rRNA sequence, or post-transcriptional modification status may predispose distinct ribosome subpopulations to preferentially engage and translate particular mRNAs. This mutual adaptation suggests a feedback mechanism by which mRNA identity can transiently co-specialize the translating ribosome, adding an additional regulatory layer atop the inherent or variant ribosomal properties that modulate translational efficiency and proteome output in a gene-specific manner. Thus, the ribosome emerges as an adaptable, programmable interpreter of the genetic code itself.

From a clinical and translational perspective, the consequences are equally far-reaching. The identification of disease-specific ribosome signatures, especially in cancers and ribosomopathies, points toward the eventual use of ribosome profiling as a new class of molecular biomarker, able to stratify patients or predict therapeutic responses based on the status of their translational apparatus [135,381]. Looking forward, the prospect of therapeutically manipulating ribosome specialization—by modulating ribosomal protein or rRNA function, installing or removing key modifications, or even deploying engineered ribosomes—holds significant promise for targeted intervention in genetic disorders, cancer, and beyond [104,359].

Emerging evidence shows that post-translational modifications (PTMs) decorate ribosome biology at multiple levels, from biogenesis to translational control and the generation of specialized ribosomes. Phosphorylation, ubiquitination and other PTMs of ribosomal proteins and associated factors are installed dynamically during ribosome assembly and on actively translating ribosomes, thereby tuning ribosome maturation, translational fidelity and elongation dynamics in response to cellular cues. Together with stage-specific rRNA modifications introduced during biogenesis, these PTMs contribute to ribosome heterogeneity and can bias defined ribosome populations toward subsets of mRNAs, supporting the concept of reprogramming ribosomes that shape context-dependent proteome output. This multilayer PTM-driven regulation adds an additional, druggable axis to the emerging “ribosome code” of gene expression control [388].

There is substantial evidence across different organisms that heterogeneous ribosome populations exist, differing in rRNA modification patterns, ribosomal protein composition, paralog usage and associated factors. At the same time, direct proof that nature routinely assembles fully specialized ribosomes “on demand” for defined transcript sets remains comparatively scarce and often context dependent. Current data are strongest for selective translational biases in specific developmental stages, stress conditions, or disease states, whereas a universal “ribosome code” is still an active area of debate. In this framework, the rationale of RiboScreenTM does not presuppose hardwired ribosome specialization, but instead builds on empirical findings that modest, targeted perturbations of individual ribosomal proteins can reproducibly bias translation of a protein of interest in yeast and human model systems.

Indeed, in part II of this review, we will describe RiboScreen^TM^ technology with empirical findings, which deploys small molecules to reprogram and specialize ribosomes for an increase or decrease in protein production level of a protein of interest in therapy, rejuvenation or biotechnology.

## 8. RiboScreen^TM^ Technology Platform Delivers Small Molecules for Precision Therapy in Rare and Prevalent Disease

Drug discovery for systemic diseases remains one of the most capital-intensive and inefficient sectors of healthcare, with failure rates approaching 90% in clinical trials [389], largely due to inadequate efficacy and toxic side effects. The root cause is poor precision in linking molecular targets to relevant biology, compounded by empirical, target-agnostic screening approaches that fail to capture the complexity of human diseases, particularly in systemic and rare disorders. Existing approaches, including molecular and AI-based screening, are still stymied by low-quality datasets and a lack of validated upstream therapeutic targets. Systemic therapies face further hurdles in safety, regulatory approval, and, in the case of rare diseases, commercial viability. Consequently, platforms that target the source of protein production—ribosome-driven translation—are emerging as a frontier for high-precision mechanistic intervention, enabling transformative strides beyond conventional protein function modulators Therefore, RiboScreen™ Platform Technology (RSPT) has opted to develop drugs that target eukaryotic ribosomal proteins to specifically gauge the protein production level of a protein of interest (POI) in a therapeutic context and in biotechnology [1,2,3,4,9].

**Figure 8:**
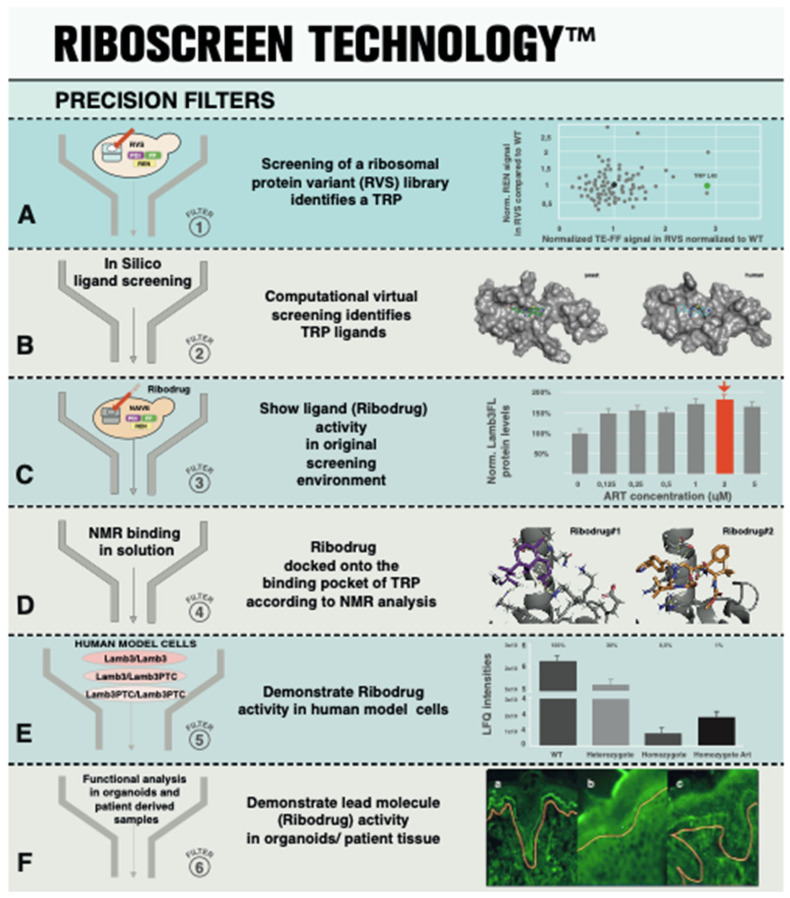
RiboScreen^TM^ platform: six-step precision filter pipeline from ribosomal target identification to functional rescue in patient tissue. The overview scheme is organized in three columns: the left column illustrates each RiboScreen^TM^ precision filter step, the middle column summarizes its functional effect on compound selection, and the right column presents representative published data visualizations supporting each filter. (**A**) First precision filter. Depicts screening of the ribosomal variant strain (RVS) library in yeast to identify a target ribosomal protein (TRP) that selectively tunes production of a protein of interest (POI) as reported by reporter luciferase signals. TRP depletion in the RVS collection reveals a ribosomal “switch” that modulates POI-FF (firefly) output while sparing the REN (Renilla control). establishing the first specificity filter [1]. (**B**) Second precision filter. Depicts AI- and structure-guided in silico screening of large compound libraries to identify TRP-directed ligands as candidate Ribodrugs [4]. (**C**) Third precision filter. Depicts functional testing of shortlisted TRP ligands in the original screening environment, the yeast RVS reporter assay, to select compounds that phenocopy the TRP depletion signature [3]. (**D**) Fourth precision filter. Depicts NMR-based structural validation of TRP–ligand interactions to confirm specific binding at an accessible ribosomal surface site [2]. (**E**) Fifth precision filter. Depicts label-free proteomic analysis of validated ligands in human model cells to demonstrate selective modulation or restoration of POI protein levels (our unpublished data). (**F**) Sixth precision filter. Depicts functional analysis in organoids and patient-derived tissue, where on the right side, (**a**) is untreated control, (**b**) is artesunate treatment for 1 month, and (**c**) is artesunate treatment for 6 months, and immunofluorescence staining of skin proteins restored by RiboScreen^TM^-guided therapy is demonstrated above the orange indication line.

Before we proceed to a detailed description of the individual steps of the RiboScreenTM platform technology, we provide a clear and self-contained description of the platform. Target selection represents the first key discriminator from conventional screens, as we first identify a ribosomal protein that acts as a translational valve on the expression level of the protein of interest (POI) we aim to modulate. The screening strategy is sophisticated in its simplicity: each POI is equipped with a luciferase tag, and a robust library of yeast vehicles harboring individual ribosomal protein depletions is screened for altered luciferase output. For validation, a unique strength of the technology is that small molecules identified as ligands for a selected ribosomal protein can be retested directly in the original yeast screening environment, confirming on-target modulation of POI levels. Specificity is then further assessed in subsequent stages of the platform, including human model cells and organoids. So far, the technology has been applied to modulate both wild-type proteins and proteins compromised by premature termination codon (PTC) mutations. For a schematic overview of the workflow, see Figure 8.

### 8.1. RiboScreen™ Technology: Why Target Ribosomal Proteins?

The number of ribosomal proteins increases only moderately across evolution—from approximately 53 in bacteria to 80 in yeast and humans—with the eukaryotic sets showing a high degree of homology. Among these, 33 ribosomal proteins are universally conserved across all kingdoms of life, while additional lineage-specific proteins have evolved to support kingdom-specific structural and functional adaptations [390].

For a structural rationale, it has to be emphasized that ribosomal proteins typically comprise a globular domain exposed on the ribosomal surface and with more extended, flexible tails embedded within the ribosomal RNA core. Critically, the extension domains of eukaryotic ribosomal proteins have expanded in conjunction with rRNA expansions, forming a cross-linked network that interconnects the functional centers of the ribosome, mRNA channel, A-site decoding center, P-site peptidyl transferase site and the exit tunnel [7]. Recent structural analyses show that evolutionary changes in ribosomal proteins preferentially occur within the extension segments, which co-evolve with rRNA expansion segments, while the globular domains remain highly conserved in structural and sequence terms [390]. Targeting these surface globular domains confers two distinct advantages. First, a minimal disruption to ribosome biogenesis, as during ribosome assembly, elongated extensions of ribosomal proteins are deeply integrated into the rRNA scaffold to stabilize subunit formation [5,390]. Drugs that specifically bind globular domains avoid interference with biogenesis, as these domains reside on the outer ribosomal surface and are spatially separated from core assembly processes.

Second, context-dependent translation editing, so that when translation-competent ribosomes are assembled on particular mRNAs, the globular domains remain accessible and modifiable. Targeting a distinct RP globular domain conceptionally allows for dynamic and mRNA-specific modulation of translation—altering parameters such as elongation rate, decoding fidelity, or termination efficiency in a manner restricted to transcripts controlled by the targeted ribosomal protein. This surface interaction can propagate allosteric changes, which “vibrate through the ribosomal functional space” [7] to functionally rewire translation mechanisms locally, while preserving global protein synthesis and cell viability—a concept supported by the allosteric network model shown by [5,7].

Targeting the globular domain of a ribosomal protein closely parallels how nature customizes translation through post-translational modifications (PTMs) of ribosomal proteins [391]. These PTMs—such as phosphorylation and ubiquitylation—are predominantly localized to the globular domains exposed on the ribosomal surface, where they serve as platforms for regulatory enzymes, envisioned as “writers,” “readers”, and “erasers”. Such strategic positioning enables dynamic modulation of translation on actively translating ribosomes, without perturbing earlier steps of ribosome biogenesis, as the flexible, extended tails of ribosomal proteins are critical for subunit assembly and stability.

From what has been said, several critical aspects of ribosomal proteins as targets for selective ribodrugs must be considered, namely the possible biochemical mechanisms that could underlie selective translational regulation; how modulation of an individual ribosomal protein might alter translation of defined mRNA subsets without broadly affecting global protein synthesis; and what structural and biophysical evidence supports allosteric communication between targeted ribosomal proteins and the ribosome’s functional centers.

Selective translational regulation can be rationalized by integrating recent insights into differential elongation kinetics with the emerging concept of ribosomal protein-mediated specialization. In agreement with our discussion in Section 6.2.6 and Section 6.2.7, multiple orthogonal approaches now demonstrate that elongation rates are not fixed but vary across individual mRNAs and physiological states, generating transcript-specific “kinetic fingerprints” of translation. These fingerprints arise from the combined influence of codon usage and tRNA supply, mRNA secondary structure within coding sequences, and nascent-chain properties such as charge and hydrophobicity, which modulate friction within the exit tunnel and the dwell times of ribosomes at defined sites. The Barna laboratory has provided the clearest evidence that altering the functional availability of ribosomal proteins can engage these pre-existing kinetic heterogeneities in a transcript-selective manner. Shi et al. [364] demonstrated that substoichiometric incorporation of distinct core RPs, such as RPL10A or RPS25, generates heterogeneous ribosome populations that preferentially translate defined subpools of mRNAs; these subsets are enriched for coherent biological programs (e.g., extracellular matrix organization, cell cycle or vitamin B12 metabolism) and exhibit distinct cis-regulatory architectures, including IRES elements in the 5′ UTR. In this framework, small-molecule binding to an individual ribosomal protein can be viewed as a specific, ligand-induced change in RP functionality that generates a new “edited” ribosome variant with altered elongation kinetics on those mRNAs whose sequence, structure or regulatory elements sensitize them to that particular RP. Such kinetic tuning can, in principle, increase or decrease protein output for a restricted transcript subset while leaving bulk translation largely unperturbed, because most transcripts do not present the local sequence/structural contexts that couple strongly to the modified RP. Although proof-of-principle data from our own and other groups support the existence of ribosome-editing small molecules with this type of transcript-selective effect, no study to date has resolved, at atomic or kinetic detail, how a specific ligand–RP interaction is transformed into a particular, genome-wide pattern of mRNA selectivity. Existing data justify the concept that ribosomal proteins are viable levers for selective translational control, but the mechanistic mapping from ligand binding to transcript-level outcome remains an open field of investigation that our RiboScreen platform seeks to address.

Recent structural and biophysical work by Timsit and colleagues now offers a compelling framework for how local perturbations at druggable RP surfaces could propagate to the core functional centers of the ribosome and thereby modulate translation in a transcript-specific fashion. Comparative structural analyses across bacteria, archaea and eukaryotes reveal that ribosomal proteins are extensively interconnected by long, often intrinsically disordered extensions that weave a dense, conserved interaction network across both subunits. These extensions make numerous tiny but topologically non-random contacts with one another and with key functional sites, including the ribosomal A-site (decoding), the peptidyl transferase center, tRNA binding sites and the peptide exit tunnel; graph-theoretical analysis shows that the architecture of this RP network has evolved so that the peptidyl transferase center and a small set of RP nodes (notably uL16, uL4, eL8 and others) have maximal betweenness centrality, i.e., they lie on most of the shortest paths for information flow [5]. In a follow-up study, Timsit et al. combined this network description with time-resolved cryo-EM to show that (i) almost all major rRNA “centers of motion” (hinges controlling subunit rotation, head swivel or stalk movements) are in direct contact with conserved RP motifs, and (ii) rRNA around the majority of RPs undergoes reproducible distance–approach (“breathing”) cycles during translocation. Acidic and aromatic residues in these motifs are positioned such that modest, context-dependent changes in electrostatic potential can switch local RNA–protein interactions from attraction to repulsion, acting as “electrostatic lever arms” that bias the motion of nearby helices and thereby tune the kinetics of tRNA movement, head/body rearrangements and channel opening at specific stages of elongation. Importantly, these RP–hinge contacts are wired into the previously described RP–RP network, which links multiple centers of motion and all functional modules into a single, highly connected graph, so that a perturbation at one RP node is expected to propagate along preferred allosteric routes towards the decoding center, PTC, tRNA A/P/E sites and exit tunnel. From the perspective of drug discovery, this implies that ligand-accessible surfaces on RPs are not isolated “dots” on the ribosomal surface, but entry points into a pre-existing, evolutionarily optimized allosteric network. A small-molecule bound to such a site can, in principle, reshape local electrostatics and dynamics at that node, alter the breathing behavior of neighboring rRNA hinges, and thereby produce subtle, topology-guided changes in the timing or amplitude of key conformational transitions during elongation. Because different mRNAs place distinct sequence features and structures into the decoding center and exit tunnel, only those transcripts whose local geometry and kinetic bottlenecks couple strongly to the perturbed allosteric pathways will experience a measurable change in elongation rate or termination behavior, producing the kind of pathway-level, transcript-subset selectivity observed for heterogeneous ribosomes and for ribosome-editing ligands in our RiboScreen studies. Together, these structural and dynamical analyses provide a coherent mechanistic rationale for how binding of small molecules to individual ribosomal proteins can be translated into selective, allosterically mediated rewiring of ribosome function, while preserving the global architecture and basal activity of the translation machinery.

RiboScreen^TM^, by acting directly on ribosomal proteins on the translating ribosome, shifts the paradigm from chasing individual protein function post-synthesis to controlling individual protein production level at synthesis—offering disease-specific, mechanistically nuanced intervention that promises increased efficacy and fewer off-target effects.

### 8.2. RiboScreen™ Platform Technology: A Stepwise Solution to Sharpen Precision in Target Validation and Drug Discovery

RiboScreen™ Platform Technology (RSPT) exemplifies a paradigm shift in drug discovery by systematically controlling the protein synthesis level of a protein of interest rather than modifying pre-existing proteins. The technology achieves its initial level of precision by targeting individual ribosomal proteins, thereby exerting direct control over protein synthesis of a protein of interest from its earliest stages. Built as an iterative pipeline, RSPT integrates molecular genetics, computational chemistry, and translational validation at successive stages. Each step functions like a sieve, eliminating compounds with low specificity or unfavorable mechanisms of action, and ensuring that only high-quality candidates progress further to functional testing in yeast and human cell models, before testing the lead molecules in organoids and animals. Consequently, RSPT is designed as a stepwise pipeline technology. This modular approach functions both as an interconnected workflow within the technology itself and as a scalable entry point into vast therapeutic spaces across disease biology and biotechnology. Below, we detail how RSPT systematically narrows promising candidates through multiple precision filters—the RSPT drug selectivity pipeline—before advancing the molecules to clinical development (Figure 8).

Step 1, the first precision filter, comprises Target Ribosomal Protein (TRP) Identification. In this initial phase, RiboScreen^TM^ technology employs as a screening tool a large collection of diploid yeast strains as a screening tool, which generates heterozygosity for individual ribosomal proteins (RPs) by heterozygous ribosomal protein gene deletions in one or the other of the 80 eukaryotic cytoplasmic ribosomal proteins, including paralogs [1]. Within this ribosomal variant protein strain (RVS) collection, each RVS carries distinct sub-populations of altered, heterologous ribosomes, demarcated by the absence of a distinct RP. Such an altered state of functional availability of a ribosomal protein serves as a proxy for testing the ribosomal protein-regulatory activity for the tailored production levels of a POI. Protein production levels are quantified through comparative protein synthesis assays, utilizing a dual luciferase assay that measures the expression level of a C-terminal Firefly (FF) tagged POI, and an independently expressed Renilla (REN) luciferase control reporter. Through rigorous statistical analysis of protein production levels observed in the RVS screening library, a variant is identified, which is demarcated by the reduced availability of the target ribosomal protein (TRP), thereby tuning the production level of the protein of interest (POI-FF), without affecting the control (REN) [1,3]. The TRP identified represents the first precision filter, a distinct ribosomal protein out of the overall set of ribosomal proteins, which is now fed into the next step of the pipeline (Figure 8A).

At this point, it is paramount to contrast conventional drug screens with those of the RiboScreen^TM^ platform technology (RSPT). In conventional drug screening strategies, the protein of interest itself serves as the bait in high-throughput assays designed to identify compounds that directly modulate its post-translational activity. By contrast, the RiboScreen™ platform technology (RSPT) operates at a more fundamental level—the translation process of the protein of interest. Once the target ribosomal protein (TRP) has been identified as the translational regulator of that protein, the next phase focuses on discovering small-molecule ligands that bind to and modulate the TRP. The objective is to convert the TRP into a precision molecular valve capable of fine-tuning the translational output of the disease-relevant protein in a controlled and reversible manner. Through this upstream and programmable intervention in protein synthesis, RSPT establishes a novel framework for therapeutic modulation that transcends the limitations of conventional post-translational drug discovery (Figure 8A).

Step 2, the second precision filter, comprises AI-Enhanced Drug Screening to deliver TRP candidate ligands. In the RSTP workflow, target ribosomal proteins (TRPs) are now employed as templates for in silico ligand discovery. Initial hit identification may arise from structure-based virtual screening, using established docking pipelines, or alternatively from literature-driven searches of previously reported ribosomal protein-interacting ligands. The selected TRP thereby functions as a molecular bait for small molecule screening campaigns, ensuring specificity at the earliest stage of evaluation.

The bioinformatic workflow begins with structural optimization of TRP three-dimensional coordinates obtained from the Protein Data Bank (www.rcsb.org). Structures are prepared using Maestro’s Protein Preparation Wizard (Schrödinger, LLC) to refine hydrogen bonding networks and assign protonation states at physiological pH, while programs such as Chimera [392] and PyMOL (Version 2.4., 2015) facilitate visualization and comparative analysis of yeast and human ribosomal proteins [393]. Potential ligand-binding sites are systematically assessed with SiteMap, and compound libraries are generated via LigPrep [394] under standardized conditions. Docking simulations are carried out with Glide [394], and candidate binding poses are evaluated according to predicted binding energies, key protein–ligand interactions, and visual inspection criteria. Cluster analysis is then applied to classify identified ligands into compound groups, for example, carboxylic acids, peptides, natural compounds, or novel chemical entities.

This bioinformatic screening process is complemented by AI-driven cheminformatics, which supports chemical space exploration, candidate prioritization, and refinement through integrated computational toxicology models, cell penetration predictions, and advanced binding simulations. Moreover, machine-learning–based predictors, such as those implemented in the AIDDISON platform, are employed to estimate Caco-2 permeability and potential cytotoxicity in HepG2 cells [395]. The combination of structural docking, predictive modeling, artificial intelligence and organic chemistry expertise thereby constitutes a second precision filter within Riboscreen^TM^, yielding TRP-directed binders with a minimized risk of off-target interactions on other ribosomal or cellular proteins. In our experience, the precision filter applied in this step of RSPT narrows the number of candidates TRP ligands down to about 50 out of more than 1 million compounds screened [4] (Figure 8B). Additional information on challenges of applying machine learning models to ribosomal proteins in virtual drug screening is reported in the Supplementary File.

Step 3, the third precision filter of the RiboScreen™ technology, focuses on functional testing in vivo cellular reporter assays of the narrowed set of putative TRP ligands. Crucially, testing of the drug candidates in wild-type yeast vehicles returns to the original RiboScreen™ screening environment to determine whether ligand binding to the TRP phenocopies the effect of TRP depletion on the production level of the protein of interest, thereby confirming that the small molecule functionally reinstates the desired translational phenotype (Figure 8C). Yeast represents an efficient and predictive model system in this context, given the strong evolutionary conservation of ribosomal protein structure and function between yeast and human cells, as well as the documented presence of identical predicted binding sites for TRP ligands on these homologous ribosomal proteins [2,4]. Testing candidate ligands in naïve yeast ribosomes thus provides a robust and cost-effective experimental strategy before transitioning to mammalian systems, enabling the detection of TRP-specific activity while minimizing unnecessary investment in nonfunctional compounds.

Candidate ligands are titrated in a broad concentration range from 1 nM to 100 µM. Molecules that only exhibit activity at higher concentrations are deprioritized because they would require extensive downstream optimization to reach efficacious nanomolar to micromolar potency. Empirical evidence has shown that typically only about one-tenth of the preselected, narrowed set of candidate pool molecules displays a significant and dose-dependent functional response. Importantly, these responsive ligands demonstrate modulation of POI_FF reporter expression selectively, while Renilla (REN) expression remains largely unaffected, confirming the specificity of their action on TRP function.

The observed changes in protein output closely parallel the effect patterns that originally defined the TRP as a ribosomal switch in the initial RiboScreen™ library screen, thereby reinforcing the functional validity of the tested compounds. In contrast, the majority of candidate molecules show little or no measurable effect on either POI_FF or Renilla reporter expression. This third precision filter thus yields a refined set of validated hit compounds capable of precisely regulating protein production levels through targeted modulation of ribosomal protein function.

Step 4, the fourth precision filter of the RiboScreen™ technology, introduces structural validation of ligand–ribosomal protein interactions through *in-solution* biophysical analysis. At this stage, the human target ribosomal protein (TRP) is engineered and optimized for nuclear magnetic resonance (NMR) spectroscopy, providing a high-resolution platform to characterize direct molecular interactions. Validated hit compounds from Step 3 are titrated against the purified TRP, and NMR spectra are recorded to monitor changes in chemical shifts that occur upon ligand binding. These spectral perturbations reflect the molecular determinants of binding strength and specificity, enabling precise mapping of ligand–TRP interaction sites in solution (Figure 8D).

A major strength of NMR is its ability to detect subtle but reproducible changes at the atomic level, thereby providing a detailed fingerprint of how small molecules engage with their protein targets. This sensitivity allows us to characterize multiple potential interaction clusters across the protein’s surface. Through careful titration and progressive monitoring of resonance shifts, amino acid residues directly participating in ligand binding can be identified with high confidence, making NMR one of the most powerful methodologies for capturing the dynamic nature of protein–ligand interactions.

In parallel, bioinformatic docking predictions derived from prior in silico analysis are systematically integrated with NMR results. By overlaying computational docking poses with experimentally observed chemical shift perturbations, the approach achieves complementary validation: computational insights guide the interpretation of NMR data, while experimental mapping narrows down predictions to the most plausible interaction clusters. In most cases, this combined strategy converges on one dominant binding cluster, corresponding to a physiologically relevant binding site that lies on a globular domain of the ribosomal protein, which is also accessible on the assembled, translation-competent ribosome [2,4].

This dual-layered interrogation of small-molecule binding—merging predictive docking with empirical structural validation—establishes a fourth precision filter in the RiboScreen™ pipeline. Compounds that pass this stage are confirmed not only as active modulators of TRP function but also as structurally validated ligands with a defined binding site. The integration of biophysical analysis and informatics modeling thus represents a decisive step in strengthening the mechanistic foundation for subsequent preclinical development.

Step 5, the fifth precision filter, of the RiboScreen™ technology represents the transition of validated hit molecules into human cell models that closely mimic disease-relevant conditions, such as protein-loss mutations or hyperactive oncogenic variants. At this stage, compounds that have successfully passed the rigorous precision filters of Steps 2 through 4 are tested directly for their impact on protein production levels of the protein of interest (POI) in a human cellular context. This shift to physiologically relevant models provides a decisive bridge toward clinical applicability (Figure 8E).

A key advantage of RSPT is that the activity of these compounds can now be assessed *without* the requirement for artificial reporter systems. Instead, endogenous protein expression is monitored directly using label-free proteomic approaches or validated through Western blot analyses. This capability significantly accelerates the evaluation process, making the workflow more resource-efficient compared with conventional drug discovery pipelines that often require extensive assay development or multiple rounds of optimization. With this direct testing strategy, lead molecules can progress into preclinical studies with markedly reduced timelines and costs.

Furthermore, a comparative proteomic analysis of untreated versus ligand-treated cells can be performed already at this step. This allows not only verification of the intended modulation of the POI, but also early detection of unwanted alterations in additional pathways that could complicate therapeutic development. By eliminating compounds that induce undesirable off-target effects, RSPT strengthens the precision of candidate advancement while minimizing downstream attrition.

This translational stage is particularly powerful in the context of drug repurposing. If a compound fulfilling the RSPT criteria is an already approved or clinically characterized medical agent, Step 5 can provide critical supportive data for exploring *off-label applications*. Demonstrating target-specific activity and functional rescue in human cell models strongly reinforces the clinical rationale for such repositioning, offering a rapid avenue from discovery to patient benefit.

Step 6, the sixth and final step, elevates the analysis to organoids, organ-on-a-chip systems, and tissue-level analogs, marking the stage at which candidate ligands are interrogated in genuinely multicellular environments. Promising compounds are examined in engineered human cell models and in artificial tissue and organoid systems that recapitulate complex, disease-relevant biology, including three-dimensional tissue architecture, multicellular interactions, and physiologically relevant gene expression environments. Within these higher-order structures, small-molecule activity can be monitored in a setting that captures both cell-intrinsic and tissue-level responses, while state-of-the-art label-free quantitative proteomics provides an unbiased readout of how TRP-targeted ligands rewire signaling and metabolic pathways across the multicellular context. High-resolution imaging—such as immunofluorescence-based quantification of proteins in human disease-state cell models and organoid sections—adds spatially resolved information, pinpointing drug-induced changes in target protein abundance or localization relative to cellular compartments and disease phenotypes (Figure 8F).

A particularly powerful dimension of Step 6 is the integration of contemporary mass-spectrometry-based proteomics, which enables large-scale pathway profiling between treated and untreated conditions in both healthy and diseased backgrounds. This approach makes it possible to systematically differentiate on-target effects from deleterious disturbances in other pathways, to identify drug-induced network perturbations, and to map molecular signatures that correlate with the desired therapeutic effect. Complementary experimental routes further broaden the functional landscape of drug activity within the same complex systems: organoid cultures derived from patient-specific stem cells and microfluidic organ-on-a-chip platforms that mimic organ-level physiology provide additional layers of translational fidelity when combined with comparative proteomics and imaging. Together, these advanced models enable a systematic exclusion of compounds with subtle yet potentially harmful side effects, while confirming that candidate ligands maintain their desired activity profile under conditions that closely approximate human tissue function.

Step 6 of the RSPT pipeline thus represents the culmination of the precision-filtering strategy. By converging proteomic pathway comparisons, high-content imaging, and complex human cell and tissue analogs, this phase delivers a finely tuned molecular portrait of drug action, thereby shortening the path to orphan-drug designation in rare diseases and compressing the preclinical phase for prevalent indications by providing decision-grade translational evidence at an earlier stage.

### 8.3. RiboScreen^TM^ Technology Prototype: Development of Artesuante to Restore Full-Length Skin Anchor Protein Lamb3 in Rare Disease Severe Junctional Epidermolysis Bullosa (sJEB)

Premature termination codon (PTC) mutations in the LAMB3 gene, encoding the critical skin anchor protein Laminin β3 (Lamb3), are the predominant genetic cause of severe junctional epidermolysis bullosa (sJEB). These mutations introduce unscheduled stop codons within the coding region, causing the synthesis of truncated, dysfunctional Lamb3 proteins. As Lamb3 is an essential constituent of the hemidesmosomal, heterotrimeric anchoring complex laminin-332 (Lm332), linking epidermis to the underlying dermis, its loss destabilizes cell-matrix adhesions across the skin and mucosal epithelia, triggering widespread epidermal blistering and internal epithelial breakdown. The resultant disruption in the basement membrane architecture leads to chronic wounds, recurrent infections, and patient death, most commonly within the first year of life [396]. Currently, there are no clinically approved therapies capable of restoring Lamb3 synthesis in sJEB. Attempts to employ aminoglycosides and other conventional readthrough-inducing drugs (TRIDs)—aimed at enabling the ribosome to bypass PTCs and produce full-length Lamb3—have failed to yield adequate efficacy, safety, or mRNA selectivity sufficient for clinical adoption [8]. RiboScreen^TM^ technology was thus leveraged to systematically discover a more targeted pharmacological strategy for the selective restoration of full-length Lamb3 [1,2,3,9].

Bauer et al., 2013 pioneered the first RiboScreen^TM^ approach by posing a central question: could modification of the ribosome itself, rather than broadly targeting core conserved functional sites of translation with conventional read-through drugs, enable selective boosting of a protein crippled by a PTC in its parent mRNA? The first technological step involved the generation and utilization of a screening library containing 137 diploid yeast strains. Each strain was heterozygous for a deletion of a single ribosomal protein gene, covering the eukaryotic cytoplasmic ribosomal proteins, including paralogous ribosomal proteins. This comprehensive collection enabled a systematic search for ribosomal proteins (RPs) acting as switches specific to increasing production levels of full-length Lamb3, encoded by a PTC mRNA, while maintaining overall ribosomal functionality and cell viability [1].

The screening platform employed co-expression of a luciferase reporter pair: (1) a human LAMB3 cDNA harboring the prevalent PTC and C-terminally tagged with firefly luciferase (LAMB3PTCFF), and (2) a Renilla luciferase mRNA as internal control (REN). These constructs featured identical regulatory untranslated regions (UTRs) derived from the yeast *ADH1* gene to ensure robust production of reporter mRNA and comparable translation initiation and termination events [1]. By quantifying reporter output across the RVS library, it was possible to systematically identify ribosomal protein alterations that specifically boosted the expression of full-length LAMB3 derived from the PTC transcript, without generally elevating global translation or bypassing PTCs of controls indiscriminately. Ribosomal protein L35 (rpL35/uL29) emerged as a compelling target: strains with altered RPL35 functional availability (depletion) displayed a robust, roughly twofold increase in full-length Lamb3 synthesis. Importantly, this boosting was indeed selective for LAMB3PTC-derived protein, while leaving production levels of a fireflyPTC (FFPTC) control reporter unaltered. This highlights rpL35 as a candidate “ribosomal switch”, a target ribosomal protein (TRP), controlling the fate of otherwise failed full-length Lamb3 production levels.

Following the identification of rpL35/uL29 as a TRP, a specificity-conferring ribosomal switch, Rathner et al. [2], in the second step of the technology, sought small-molecule binders of rpL35/uL29 that could allosterically modulate rpL35/uL29 to mimic the genetic partial depletion observed in the RVS screening tool. Rather than screening vast compound libraries with undirected high-throughput approaches, the team pursued a rational, structure-informed strategy based on a literature search. Indeed, previously, in an unrelated context, a putative small molecule binder of rpL35/uL29 was reported, artesunate [397]. The authors then subjected L35 to molecular docking simulations, leveraging solved cryo-EM structures of human and yeast ribosomes. Notably, artesunate (a well-tolerated antimalarial) and atazanavir (an antiretroviral protease inhibitor), both of which are FDA-approved drugs, emerged as predicted L35 binders, based on favorable ΔG binding energies and drug-like physicochemical properties [2]. Notably, the predicted binding clusters for these drugs overlapped in the N-terminal region and helix 2 of rpL35/uL29, an area accessible on the ribosome’s surface [2]. In the next technology step, to experimentally confirm the predictions, recombinant human rpL35/uL29 was expressed in *E. coli*, isotopically labeled, and purified to homogeneity, enabling NMR titration studies. The addition of artesunate and atazanavir to labeled L35 generated clear, concentration-dependent chemical shift perturbations in 1H,15N HSQC spectra—validating direct binding in solution. Furthermore, a combination of NMR and molecular docking data revealed overlapping yet distinct binding pockets for artesunate and atazanavir in the aforementioned N-terminal cluster—providing orthogonal, tangible sites on rpL35/uL29 for pharmacological manipulation [2].

In a subsequent step, Wimmer et al. [3] pursued essential translational and functional validation of these findings by treating naive yeast strains co-transformed with the dual-luciferase LAMB3PTCFF/REN system—the original screening environment—with artesunate and atazanavir. A rigorous dose–response design revealed that both artesunate and atazanavir, when added to yeast cultures for 18 h, selectively increased full-length LAMB3 biosynthesis by up to twofold, mirroring the results from the original genetic screen. Critically, this effect was also highly selective: Artesunate and atazanavir had no effect on the expression of a control full-length firefly luciferase (FFFL) derived from a PTC-containing mRNA, ruling out a general non-specific PTC readthrough activity. Furthermore, erythromycin, a macrolide antibiotic known to disrupt bacterial ribosomal elongation and to act as a general PTC readthrough drug [331,398], was used as a positive control. As anticipated for a general readthrough enhancer, erythromycin boosted both full-length LAMB3 and FFFL, contrasting strongly with the selectivity of artesunate and atazanavir. Perhaps most intriguingly, low-dose combination treatment with artesunate and atazanavir achieved equivalent full-length Lamb3 (protein) boosting at significantly reduced individual drug concentrations, supporting their cooperative, synergistic action and suggesting the binding pockets could be simultaneously targeted for future therapeutic optimization [4].

As an essential step for translational relevance, the next technology step shifted from yeast to human disease models. The study by Moßhammer C., 2021 [399] describes the generation of human keratinocyte models bearing the LAMB3R635X variant—both in the heterozygous “carrier” and homozygous “patient” state, using precise CRISPR/Cas9 gene editing. Comparative quantitative proteomics established that, relative to wild-type parental cells, the basal level of full-length LAMB3 in homozygous mutants was a mere 0.5%, aligning with the clinical reality of catastrophic protein loss in sJEB. Treatment of these homozygous patient-model cells with artesunate for 18 h induced an approximate twofold increase in full-length LAMB3 protein (to 1% of wild-type), thereby reaching the predicted threshold for partial functional correction based on prior genotype–phenotype observations [8,9]. The robust selectivity and reproducibility of this response in a human cell context further validate RiboScreen^TM^-guided rpL35 targeting as a prototype for disease-specific ribosome editing intervention. To bridge in vitro–to–in vivo studies, Sinz et al., 2025 provided an additional pivotal insight. By immunofluorescence, they demonstrate that both the CRISPR-engineered model keratinocytes and also immortalized patient-derived keratinocytes respond to artesunate (72 h treatment) with clear, quantifiable increases in full-length Lamb3 expression [9]. This time-dependent accumulation underlines the possibility of steady, therapeutically meaningful Lamb3 restoration with repeated or sustained drug exposure. Collectively, these studies promoted the first compassionate off-label clinical use as a first-in-man study of artesunate in an infant with sJEB. This documented the drug’s tolerability (administered intravenously, orally, and topically), and, after several months’ treatment, partial wound healing at previously intractable sites.

In sum, we have substantiated the claim of the therapeutic potential of artesunate by a series of complementary assays and methodologies. Building on an in vivo translational assay in yeast, we first showed that artesunate selectively boosts expression of full-length Lamb3 encoded by LAMB3-PTC mRNA up to two-fold, while control reporters remain largely unaffected [3]. This mechanistic selectivity was then translated into human systems: immunofluorescence analyses in two independent keratinocyte models (sJEB-LAMB3PTC HaCaT cells and immortalized patient keratinocytes) demonstrated a significant artesunate-induced increase in full-length laminin β3 signal after short-term treatment [9]. These findings are mirrored by label-free proteomics in human model cells, where artesunate treatment leads to an approximate two-fold increase in full-length Lamb3 [399]. Together, these converging preclinical data, complemented by first safety and efficacy observations from off-label artesunate use in an infant with sJEB, provide a coherent preclinical–clinical chain of evidence for therapeutic translation.

While the clinical outcome was ultimately limited by factors such as drug bioavailability and advanced disease state, the observed improvement in epithelialization provides a crucial proof-of-concept for artesunate’s mechanism, safety, and future optimization as a repurposed or next-generation therapeutic [9]. This study also paved the way for future development of Artesunate derivatives to provide superior molecules for the treatment of sJEB.

#### 8.3.1. Artesunate Versus Conventional Drugs in Restoring Full-Length Protein Production from a PTC mRNA

In our pilot and prototype study, we investigated the small-molecule Artesunate that acts by selectively editing a ribosomal protein to restore production levels of the full-length skin anchor protein Lamb3, compromised by a premature termination codon (PTC) in its parental mRNA [1,2,9]. To illuminate the significance of this finding, it is useful to first consider how conventional translational readthrough-inducing drugs (TRIDs) operate. We will discuss conventional TRIDs, including aminoglycosides, macrolides, and ataluren, which all, by different mechanisms, change fidelity of A-site decoding to promote incorporation of a near cognate tRNA, thereby enabling synthesis of full-length proteins. We then contrast this established paradigm with the distinct concept of ribosome editing through ribosomal protein targeting. Although we initially describe this mechanistic framework in the context of a PTC-containing transcript, the fundamental principle can be broadened to other cases of selective mRNA translation control, applicable not only to transcripts harboring disruptive mutations but also to native mRNAs or those carrying hyperactive sequence variants.

The canonical route for restoring full-length protein from a PTC-containing mRNA builds on the fact that translational fidelity is not absolute [268,400,401,402,403]. Even under physiological conditions, a basal level of readthrough occurs when, on rare decoding events, a near-cognate tRNA is misincorporated at the A-site of the ribosome, thereby permitting translation to continue beyond the premature stop codon. This naturally occurring mechanism, termed basal readthrough, however, is highly inefficient and produces only trace amounts of full-length protein [404,405,406,407]. Conventional TRIDs—such as aminoglycosides, macrolides, and ataluren—stimulate this same principle by enhancing near-cognate tRNA acceptance at the ribosomal A-site. Aminoglycosides directly bind to the ribosomal A-site, reducing its fidelity for all decoding steps and thereby increasing the probability of near-cognate tRNA incorporation. In a series of excellent studies, the mode of action of aminoglycosides has been determined for bacterial ribosomes, the target for classical antibiotic treatment [408]. Aminoglycoside antibiotics such as gentamicin, tobramycin, and netilmicin exemplify a classic group of translational readthrough-inducing drugs that exert their effects by binding predominantly to helix 44 (h44) of the bacterial 16S rRNA [409]. The primary interaction site in bacterial 16S rRNA is defined by two universally conserved adenine residues, A1492 and A1493. In eukaryotes, including yeast and humans, A1755 and A1756 of the 18S rRNA are the homologous decoding-center residues corresponding to bacterial A1492 and A1493, respectively [410]. During cognate decoding in bacteria, these two adenines flip out of h44 and clamp the codon–anticodon minihelix at the first two positions of the codon, while the third position remains more permissive and accommodates wobble pairing. When aminoglycosides bind in the groove normally occupied by the two adenines, they stabilize A1492 and A1493 in an extrahelical conformation even in the absence of perfectly matched codon–anticodon pairing [409]. This conformational shift compromises the ribosome’s intrinsic proofreading function at the A site, facilitating the accommodation of near-cognate aminoacyl-tRNAs (aa-tRNAs) regardless of codon–anticodon accuracy. As a consequence, the ribosome misincorporates incorrect amino acids into nascent polypeptide chains at both sense and stop codons, generating aberrant proteins that are frequently nonfunctional or deleterious, thereby disrupting cellular processes and ultimately contributing to bacterial cell death. When aminoglycosides are repurposed to treat premature termination codon (PTC) disorders in humans, their fundamental mode of action is retained, but the detailed molecular interactions are modulated by differences in eukaryotic ribosomal architecture. The aminoglycoside-binding pockets in human (and yeast) 18S rRNA are considerably shallower and chemically distinct compared to those in bacterial 16S rRNA, resulting in reduced drug affinity and a correspondingly dampened disruption of translational fidelity [410,411,412,413]. However, although this lower affinity limits the frequency and severity of miscoding relative to bacterial ribosomes, aminoglycosides still induce substantial misreading in human cells, affecting not only PTC sites but, in principle, any sense codon across the transcriptome [316,414]. Thus, their application enhances PTC readthrough relative to basal cellular mechanisms but is intrinsically accompanied by pervasive, non-selective translational errors.

Macrolides, such as erythromycin, act from a different ribosomal locus, binding within the exit tunnel adjacent to the peptidyl transferase center. By obstructing or modulating the path of the nascent peptide, they alter elongation dynamics and the functional properties of the catalytic core [415,416,417]. Importantly, these effects are not confined to the tunnel itself: through long-distance allosteric communication within the ribosome, macrolides perturb the decoding machinery at the A-site, indirectly lowering the accuracy of tRNA selection [415,418]. This coupling between spatially distant ribosomal domains highlights the ribosome’s integrated structural network, where local perturbations reverberate into the decoding center. Like aminoglycosides, macrolides have long been exploited in clinical antibiotic therapy, capitalizing on their robust misreading of mRNAs in bacterial translation. However, while differences between bacterial and human ribosomes reduce their toxic potential in human cells, their inherent lack of mRNA specificity and capacity to affect general decoding fidelity mean that they, too, cannot serve as selective readthrough-inducing agents in humans without severe off-target consequences [419,420,421,422].

Ataluren represents a mechanistically distinct case. Instead of binding to a defined ribosomal functional site, its mode of action appears to involve delaying translation termination by interfering with the activity of the release factor complex. This kinetic stalling extends the opportunity for a near-cognate tRNA to be sampled at the A-site, thereby allowing elongation to continue past premature stop codons. While often presented as comparatively selective, the underlying process is still rooted in modulating A-site behavior, and, by extension, is not inherently transcript-specific. Clinical studies highlight a more favorable safety profile for ataluren compared to classical antibiotics, yet response rates remain inconsistent, and its indiscriminate influence on stop codon recognition raises the possibility of unintended perturbation at normal termination sites or regulatory premature stop signals [404,423,424,425].

Conventional translational readthrough-inducing drugs (TRIDs) thus share a unifying feature: they act upon ancient, conserved structural hubs of the ribosome and thereby exert their effects indiscriminately across all mRNAs undergoing translation. Although their molecular entry points differ, each drug class ultimately converges on the ribosomal A-site—the central decoding hub where codon–anticodon recognition occurs and where near-cognate tRNAs can, under reduced fidelity, be accommodated. This convergence explains their ability to promote readthrough at premature termination codons, but also underlies the non-selectivity and off-target effects that limit their sustained application in humans [310,413,414,426].

#### 8.3.2. The Mechanism of Action of Drugs Turning Ribosomal Proteins into Molecular Valves for Customized Change in Protein Production Levels

To elucidate the selective modulation of protein production by targeting ribosomal proteins, it is essential to first introduce the role of translation elongation as one of the principal determinants in the control of protein synthesis output. For decades, elongation rates were widely thought to be invariant across cell types and conditions, with only sporadic experimental evidence suggesting otherwise. However, emerging work has brought to the forefront the decisive role of elongation speed as a major player in modulating protein synthesis rates, demonstrating that changes in elongation can dramatically alter the cellular proteome in both qualitative and quantitative terms [270,297,427,428]. The study by Gerashchenko et al. 2021 was the first to directly measure translation elongation rates in living mammals using quantitative ribosome profiling. In this pioneering work, mean elongation rates were quantified as 6.8, 5.0, and 4.3 amino acids per second in mouse liver, kidney, and skeletal muscle, respectively. Although this is a single comprehensive study and further research may extend this physiological range, these results suggest that elongation rates in mammalian tissues can vary by more than 50% across organs. Given potential biological and technical variability, it can be assumed that elongation rates may span at least from about twice the mean down to half the mean up—indicating an expected range of approximately two-fold variation from a given organ-specific baseline [295,297].

In this context, the dynamic contributions of ribosomal proteins to the regulation of elongation must also be recognized as central to this process. Contemporary studies from several laboratories, including ours [3,4,163,268,429,430,431], converge on a paradigm shift: ribosomal proteins are not passive structural components of the ribosome but dynamic regulators of translation, capable of conferring transcript-specific specialization to the ribosome. Modifying or selectively targeting a ribosomal protein—whether through post-translational modification or small-molecule interaction—can generate a functionally distinct ribosome variant with specialized properties and translation kinetics. Importantly, these specialized ribosomes can modulate elongation in a context-dependent manner, establishing a form of ribosomal “precision tuning” that affects only one mRNA species or a defined subset of transcripts, while preserving global protein synthesis. Small molecules acting as “ribosome editors” exemplify this concept. By binding to surface-exposed domains of specific ribosomal proteins, they can act as molecular valves that fine-tune elongation velocity only when the ribosome engages an mRNA with a particular sequence or structural context [354]. The functional sensitivity of the affected mRNA is dictated by the local geometry of the ribosome–mRNA interface, meaning that even subtle alterations in ribosomal protein conformation can result in transcript-selective control of translation output. Through this mechanism, only defined groups of transcripts—for example, those with complex secondary structures or unique codon contexts—are significantly influenced, while bulk cellular mRNAs remain unaffected [432,433,434]. Importantly, this selectivity arises from the way in which the modified ribosomal protein influences local decoding kinetics during elongation of its “companion” mRNA. Thus, whether an mRNA is wild-type or carries a PTC is irrelevant for the specialized ribosome generated by ribosomal protein editing. However, the consequences for protein synthesis will diverge dramatically depending on the translational landscape of a wild-type mRNA or its PTC derivative.

A compelling illustration of this principle is provided by the work of Wimmer et al., 2024, who studied LAMB3, a gene encoding a skin-anchoring protein often disrupted by PTC/nonsense mutations. They investigated whether a small molecule, artesunate, acting on a single ribosomal protein (rpL35/uL29), could adjust translation of both the wild-type and the PTC-containing LAMB3 mRNAs. Remarkably, artesunate treatment caused a modest decrease in the output of wild-type LAMB3 protein, but simultaneously produced a twofold increase in the full-length protein derived from the PTC-containing LAMB3 transcript. This dual effect indicates that artesunate’s action is not a general enhancement of readthrough but a context-dependent response shaped by the specific interaction between RpL35-modified ribosomes and LAMB3 mRNA. Control experiments using FF luciferase and its PTC variant confirmed that this regulation was exclusive to the LAMB3 transcript environment [3].

Mechanistically, artesunate binding to rpL35/uL29, hitherto rpL35, appears to reduce the elongation rate of ribosomes translating LAMB3 mRNA. For the wild-type transcript, this slowdown modestly decreases the synthesis rate. However, for the PTC-containing mRNA, the same kinetic slowdown prolongs the ribosomal dwell time at the stop codon, increasing the likelihood that a near-cognate tRNA is accepted at the PTC codon, instead of triggering termination, thereby restoring full-length protein production. This inverse relationship—where a decrease in wild-type protein output accompanies an increase in full-length protein from a PTC-containing derivative—illustrates the principle of kinetic selectivity. By adjusting local decoding dynamics rather than applying a global readthrough stimulus, artesunate accomplishes selective rescue of a defective protein without disrupting overall translation fidelity.

The guiding principles underlying this strategic selectivity echo notions first articulated by Francis Crick in the late 1950s. Crick speculated that ribosomes might exhibit specialization—at the extreme, a “one gene–one ribosome–one protein” model [343]—which was later discounted in its absolute form but inspired decades of research into ribosome heterogeneity and specialization. The emergence of the ribosome code model—where variant ribosomes, defined by unique modification of a ribosomal protein or rRNA composition or modification, govern translation for selected mRNAs—brings Crick’s conceptual insight into modern translational medicine. Small molecule ribosome modulators, by targeting the versatile, surface-exposed ribosomal protein domains rather than the universally conserved rRNA core, now enable a regulatory precision that reshapes both therapeutic potential and our understanding of gene expression regulation.

### 8.4. Boosting Production Levels of Tropoelastin—Precursor of Skin Protein Elastin

The RiboScreen platform represents a transformative advance in molecular medicine, with demonstrated capability far beyond a therapeutically most important, but overall narrow focus on premature termination codon (PTC) mutation rescue. Instead of being exclusive to restoring the translation of mutant PTC mRNAs, RiboScreen^TM^ Technology enables the customized protein output—boost or reduction—of any protein of interest (POI), regardless of coding sequence status. This versatility was recently exemplified in its application to tropoelastin, the soluble precursor of elastin, a protein essential for tissue elasticity in skin, blood vessels, and tendons [4,435]. Unlike our previous approach centered around PTC mutation rescue, the study focused on boosting wild-type tropoelastin, leveraging engineered translational control of protein production levels for regenerative and cosmetic medicine. The biological rationale is potent: elastin turnover decreases with age, and restoring its precursor tropoelastin at physiological levels holds promise for skin rejuvenation, wound healing, and supplementation of elastic tissue function [4].

Employing the RiboScreen^TM^ precision filter pipeline, a ribosomal protein that acts as a dedicated translational switch for tropoelastin was identified using the genetically defined yeast library in combination with a dual luciferase reporter assay. Here, strains were co-transformed with two companion reporters: a Firefly luciferase fused C-terminally to human tropoelastin isoform 6 (TE-FF) and an independently expressed Renilla luciferase control (REN), both driven by identical ADH1-derived 5′ and 3′ UTRs to ensure matched initiation and termination characteristics.

Luciferase outputs for TE-FF and Renilla were recorded in each ribosomal variant strain and normalized to wild-type levels, followed by rigorous outlier elimination and descriptive statistics. To condense the dual-reporter output into an interpretable screen, the mean normalized TE-FF signal for each strain was plotted against the corresponding normalized Renilla signal in a correlation plot, effectively visualizing how each individual ribosomal protein perturbation redistributed production of the tropoelastin reporter versus the control protein. Most strains clustered within a narrow region around unity for both reporters, indicating that partial depletion of the majority of ribosomal proteins neither specifically affected tropoelastin translation nor globally disturbed bulk protein synthesis.

Within this global pattern, a single ribosomal variant strain emerged as the desired hit: heterozygous depletion of rpL40, encoded by RPL40A, produced a selective 2.7-fold increase in TE-FF reporter expression, while REN levels remained indistinguishable from wild type. Other candidate variants showed increased TE-FF but concomitant changes in Renilla and were therefore excluded as non-specific. In sum, rpL40 emerged as the target ribosomal protein (TRP) that selectively boosts tropoelastin translation in a physiologically moderate (two- to four-fold) range, providing a mechanistically defined entry point for subsequent ligand discovery.

In the next RiboScreen^TM^ precision step, Bioinformatics-Led Ligand Discovery for eL40 was performed. Having defined rpL40/eL40 as a translational regulator, the next phase employed bioinformatics and cheminformatics for ligand screening on yeast and human eL40. Using tools such as UCSF Chimera, the three-dimensional structure of eL40 from yeast and human ribosomes was visualized and analyzed to evaluate Coulomb surface potentials, revealing candidate regions compatible with ligand binding—specifically, a central domain lined by basic amino acids and unshielded by rRNA in the assembled ribosome [4]. SiteMap (Schrödinger) was used to systematically identify small pockets with possible druggability near key residues (Asp92 in humans, Ser94 in yeast), generating a shortlist of accessible binding sites. While their scores suggested moderate drug ability, they formed adjacent and potentially dynamically connected pockets suitable for small molecule docking on yeast and human eL40 [3]. This was followed by virtual Screening and Docking of Large Compound Libraries.

Leveraging the insights of pocket identification, a subset of Merck’s in-house chemical library underwent virtual screening using Glide docking. Hits were prioritized based on docking scores, pose proximity to critical residues (alanine 107 and aspartate 92 of rpL40/eL40), and visual inspection of ligand orientation within the eL40 pocket. Out of hundreds of candidates identified, 33 molecules were shortlisted—spanning carboxylic acids, peptides, natural compounds, and novel chemical entities [4].

The next RiboScreen^TM^ precision step was experimental validation of candidate rpL40 ligands in cellular assays. A total of 29 available candidate ligands were tested in the original screening environment, using naïve yeast vehicles co-expressing the TE-FF and Renilla reporters. Using the Promega Dual Glow Assay, dose-dependent effects were measured across concentrations from 1 nM to 100 µM. The standout hit, compound 17 (C17), delivered a robust 1.7-fold increase in tropoelastin reporter levels at 100 µM without affecting REN, closely mirroring the effect seen with genetic depletion of eL40. Further structural analysis revealed favorable binding interactions: a phenolic group forming hydrogen bonds with alanine 107 and a piperidine moiety engaging aspartate 92 through charge-based contacts—features consistent across yeast and human orthologs [3].

Other compounds, including C7 and C25, exhibited various degrees of efficacy, while the majority did not alter either reporter signal, underscoring the specificity of the platform’s ligand screening approach [3]. Testing the activity of rpL40 ligands in a human model will be performed in further studies.

### 8.5. RiboScreen^TM^ Technology—Outlook for the Pipeline

RiboScreen^TM^ Technology is positioned at the forefront of precision medicine, with an exceptionally broad horizon for pipeline development. The platform is designed to address the pressing limitations of current drug discovery by leveraging targeted ribosomal modulation to precisely control protein production—for PTC targets, for “undruggable” targets and across virtually any disease-relevant protein in human biology.

#### 8.5.1. Expanding Therapeutic Horizons for PTC-Associated Rare Diseases Through RiboScreen Technology

Premature termination codons (PTCs) account for a substantial fraction of 11% of pathogenic variants across monogenic rare diseases, making them an attractive, genetically defined target class for precision therapeutics. By restoring full-length protein from PTC-containing alleles, pharmacological readthrough has emerged as a unifying strategy across otherwise distinct indications, but existing agents, collectively termed translation read-through-inducing drugs, TRIDs, such as aminoglycosides and ataluren, are limited by a lack of selectivity, variable efficacy, and narrow therapeutic windows [316,322,436,437]. Against this backdrop, the RiboScreen^TM^ platform is positioned as a next-generation approach that systematically identifies highly specific ribosome-modulating molecules with the potential to correct nonsense mutations across multiple rare diseases while minimizing off-target effects.

In severe junctional epidermolysis bullosa (JEB) [396,438,439] and recessive dystrophic epidermolysis bullosa (RDEB) [440,441], PTC/nonsense mutations in structural skin proteins such as laminin-332 or type VII collagen led to extreme skin fragility, chronic wounds, and high morbidity. Proof-of-concept for PTC readthrough in EB has been provided by intravenous gentamicin, which restored type VII collagen (C7) expression, promoted wound closure, and improved clinical scores in small RDEB cohorts, without major ototoxic or nephrotoxic adverse events over short courses (clinical trials, NCT (National Clinical Trial), NCT02698735; NCT03392909), described in [442]. Nonetheless, aminoglycoside therapy remains constrained by batch-to-batch variability in readthrough potency, systemic toxicity concerns, and non-selective effects on global translation, motivating the search for safer, more targeted modulators [443]. A platform such as RiboScreen^TM^, which can be tailored to specific PTC contexts and protein outputs, could substantially extend the therapeutic options for JEB and RDEB, including patients ineligible for gene or cell therapies.

Cystic fibrosis (CF) due to PTC/nonsense mutations in the *CFTR* gene (Cystic fibrosa transmembrane receptor) is another area where TRIDs readthrough approaches have been clinically explored. Ataluren (PTC124) reached phase 2 and 3 trials in CF, where it increased production of full-length CFTR protein and showed pharmacological activity in subsets of patients, though overall clinical benefit proved modest and context-dependent (NCT00803205; NCT02139306) [444,445,446]. Gentamicin and related aminoglycosides likewise demonstrated restoration of CFTR function in preclinical and early clinical studies but were hampered by toxicity and variable responses, including those due to differences in PTC sequence context (clinical trials, NCT00376428; NCT02965326; NCT00458341; NCT00351078), described in [447]. These experiences underscore how codon identity, local sequence context, and tissue-specific translation dynamics critically shape readthrough outcomes, supporting a shift from “one-size-fits-all” small TRIDs molecules toward platforms that can screen and optimize for disease- and mutation-specific ribosomal responses, as envisioned with RiboScreen^TM^ Technology [9].

In Duchenne muscular dystrophy (DMD) caused by PTC nonsense mutations in dystrophin, a protein acting in muscle contractility, ataluren has advanced furthest, becoming the first approved small-molecule readthrough therapy for nmDMD (nonsense mutation DMD) in several jurisdictions, based on data showing increased dystrophin expression and trends toward slower functional decline (clinical trial, NCT01826487), described in [448,449]. Ongoing and recent phase 2 and extension studies with ataluren continue to refine its benefit–risk profile and durability of effect, while aminoglycoside-based strategies remain largely constrained to experimental or early-phase settings because of safety limitations. These clinical programs validate PTC readthrough as a disease-modifying principle in DMD but also highlight its incomplete efficacy, leaving room for more potent and specific ribosome-modulating compounds that could be identified using RiboScreen^TM^ to enhance dystrophin rescue across a broader patient subset [322,450].

A rationally guided ribosome-centric discovery platform could systematically identify small molecules or combinations that achieve sufficient, tissue-appropriate protein restoration for other PTC indications as well, for example, PTC/nonsense variants of CFTRR (cystic fibrosis transmembrane conductance regulator) in cystic fibrosis [444,445] or PTC mutations in Rett syndrome. Rett syndrome provides a particularly challenging example, as a sizeable proportion of methyl-CpG-binding protein 2 (MECP2) mutations are PTC/nonsense variants, but the therapeutic window for MECP2 expression is narrow, and both under- and overexpression are deleterious. Ataluren has been evaluated in preclinical Rett models with mixed and often controversial results, and concerns have been raised about its precise mechanism of action and reliability as a readthrough agent in this context [451,452]. These limitations underscore the need for platforms capable of finely tuning translational outcomes at gene-specific PTCs and rigorously separating bona fide readthrough from off-target or reporter-specific effects, which is central to the design philosophy of RiboScreen^TM^ Technology.

#### 8.5.2. Expanding Therapeutic Horizons for PTC-Mutated Tumor Suppressors Through RiboScreen^TM^ Technology

Nonsense mutations in tumor suppressor genes create premature termination codons (PTCs) that truncate key regulatory proteins and disable critical barriers to malignant transformation. Around 10% of TP53 mutations are nonsense variants, representing a major genetically defined subpopulation of cancers that lack effective, targeted therapies [453,454,455,456,457]. Unlike Mendelian disorders, where partial restoration of protein function in a single tissue can be sufficient, oncology requires sustained, tumor-selective rescue of tumor suppressor activity without globally disturbing translation, underscoring in this therapeutic area also the need for more selective therapeutic options than current readthrough drugs can provide [322,454,457].

TP53 is the most frequently mutated tumor suppressor in human cancer, and nonsense mutations in TP53 alone account for roughly one million new cancer cases annually worldwide [453,458,459,460]. Preclinical studies have shown that aminoglycoside antibiotics such as G418 and gentamicin can induce translational readthrough of multiple TP53 nonsense mutations, including common variants like R213X, R196X, and R306X, leading to detectable full-length p53 protein [454]. For the prototypical R213X mutation, aminoglycoside-induced readthrough yields functional p53 capable of inhibiting tumor-cell growth and triggering apoptosis, confirming that the restored protein retains canonical tumor suppressor functions. Gentamicin B1, a minor component of clinical gentamicin preparations, has been identified as a particularly potent p53 PTC readthrough agent at R213, achieving robust suppression at all three stop codons at this position, although the original report was later retracted and remains under discussion, highlighting the fragility of the evidence base [454].

Beyond aminoglycosides, several non-antibiotic compounds have emerged as TP53 readthrough candidates. The chemotherapeutic 5-fluorouracil, through its metabolite 5-fluorouridine (FUr), induces translational readthrough of the TP53 R213X, with ribosome profiling confirming production of full-length p53 in tumor cells. High-throughput screens have also identified small molecules that synergize with G418 or with eRF3 degraders to enhance readthrough of TP53 and PTEN nonsense mutations, suggesting combination regimens as a route to lower aminoglycoside doses while maintaining efficacy [461]. Ataluren (PTC124), a well-known clinical readthrough agent, has been widely studied in genetic diseases and evaluated in cellular TP53 models, but it has not advanced into late-stage oncology trials specifically targeting TP53 nonsense mutations, in part due to inconsistent readthrough activity and mechanistic uncertainties [454,461].

To date, translational readthrough strategies for TP53 remain largely in the preclinical domain, with no approved therapies and only very limited early-phase clinical exploration in cancer patients [454]. The main barriers are well recognized: aminoglycosides like G418 and gentamicin are associated with significant nephrotoxicity and ototoxicity, making chronic systemic administration for cancer treatment problematic, and their lack of codon and gene specificity raises concerns about global perturbation of translation termination. Moreover, not all TP53 PTCs respond equally; sequence context around the stop codon critically shapes readthrough efficiency, resulting in heterogeneous rescue even within the same gene. These constraints strongly argue for discovery platforms that can tailor small-molecule responses to individual nonsense mutations with a target mRNA rather than relying on a narrow set of broadly acting antibiotics [454,461,462,463].

Other tumor suppressor genes harbor clinically relevant PTCs that are, in principle, amenable to pharmacologic readthrough. In BRCA1, nonsense mutations such as R1751X lead to loss of homologous recombination repair and high breast and ovarian cancer risk; preclinical work has demonstrated that G418 treatment can induce BRCA1 PTC readthrough and restore functionally relevant levels of full-length BRCA1 protein, re-establish DNA repair capacity, and rescue cell-cycle checkpoint function in BRCA1-mutant tumor cells: Again, readthrough efficiency are strongly influenced by local sequence context and these findings so far come from in vitro models using an aminoglycoside with a limited safety profile for clinical use [464].

The APC tumor suppressor, frequently inactivated in colorectal cancer, carries recurrent PTCs such as R1450X that truncate the protein and hyperactivate Wnt signaling; aminoglycosides and some macrolides have induced readthrough of APC PTCs in cell models, partially restoring full-length APC and inhibiting growth of APC-mutant colorectal carcinoma cells [463,465]. RB1, encoding the retinoblastoma protein, is another prominent example: approximately 26% of somatic RB1 mutations are PTC/nonsense substitutions, and recent work has shown that G418 can induce readthrough of RB1 PTCs, increase full-length protein levels and reduce viability of RB1-mutant cells.

Collectively, these examples underscore both the conceptual validity and the practical limitations of using TIRDs to rescue tumor suppressor function. Aminoglycosides, FUr, and related agents demonstrate that pharmacologic suppression of PTCs in TP53, BRCA1, APC and RB1 is feasible, but their toxicity, off-target effects, and mutation-specific variability have so far prevented broad clinical adoption in oncology. A platform such as RiboScreen^TM^, designed to interrogate ribosome behavior for PTCs of a given mRNA and to discover highly selective, context-optimized modulators, offers a route to overcome these barriers by uncoupling therapeutic readthrough of tumor suppressor PTCs from global disruption of translation, thereby expanding the realistic therapeutic scope for TP53-centric interventions and for other key tumor suppressors in cancers driven by nonsense mutations.

#### 8.5.3. Expanding Therapeutic Horizons for Selective Downregulation of Hyperactive Oncoproteins Through RiboScreen^TM^ Technology

Directly suppressing hyperactive oncogenes at the level of protein synthesis offers a complementary strategy to existing attempts to inhibit their activity or promote their degradation. RiboScreen^TM^ frames this as a translational control problem: instead of chasing difficult binding pockets or complex protein–protein interfaces, it aims to modulate ribosome translational activity on oncogenic mRNAs encoding paradigmatic undruggable oncoproteins—among them an intrinsically disordered transcription factor oncogene and an oncogenic small GTPase—thereby reducing oncoprotein abundance at its source.

The paradigmatic intrinsically disordered transcription factor oncogene represents a quintessential undruggable target, largely because its structural disorder precludes well-defined small-molecule binding pockets, yet modest increases in its expression are sufficient to drive tumor initiation and maintenance. Therapeutic approaches have therefore focused on indirect suppression via epigenetic and cell-cycle kinase inhibitors that downregulate its transcription, interference with oncogenic transcription factor–co-factor dimerization, or targeted protein degradation [466,467,468,469]. A dual bromodomain/histone deacetylase inhibitor reached phase I/II trials (NCT02674750) and showed anti-tumor activity in cancers driven by this disordered transcriptional driver, but its effects are broad, with toxicity and resistance emerging as important limitations [470]. More recently, oncoprotein-directed PROTAC degraders and allele-specific degraders targeting this transcription factor have entered early-phase clinical evaluation (NCT05100251, NCT04808362), providing the first proof that direct degradation of this oncoprotein is pharmacologically feasible in patients, although long-term efficacy and safety remain to be defined. Preclinical work also highlights important complexities: certain PROTAC degraders targeting this disordered oncogene can generate N-terminally truncated protein species that retain oncogenic activity, illustrating how protein-level interventions can carry unanticipated consequences [471,472].

At the translational level, this intrinsically disordered transcriptional driver appears particularly vulnerable. Recent data show that ribosomes stall during synthesis of their N-terminal portion, requiring a dedicated ribosome-associated quality control sensor to resolve stalled translation and maintain oncoprotein levels. Genetic perturbation of these quality control components selectively impairs tumor growth driven by overexpression of this disordered oncogene in glioblastoma and other models, demonstrating that cancers reliant on its elevated abundance depend on finely tuned translation and mRNA quality control [473]. These findings open an attractive window for platforms such as RiboScreen™, which could identify small molecules that exaggerate or reprogram this stalling response specifically on the corresponding transcripts, lowering oncoprotein output without broadly suppressing global translation. Critically, the structural disorder that renders this transcription factor resistant to conventional inhibition is precisely the property that makes its translational synthesis a compelling intervention point: there is no active site to mutate, no alternative folded scaffold to exploit, and no bypass mechanism available at the ribosome level.

The oncogenic small GTPase family has long represented another quintessential category of undruggable targets. Decades of effort to inhibit upstream or downstream signaling achieved only partial success, mainly because pathway feedback and redundancy rapidly restore GTPase-MAPK signaling. The breakthrough came with mutant-allele-specific covalent inhibitors targeting the most prevalent glycine-to-cysteine substitution in this GTPase family (Clinical Trials, NCT04185883; NCT05014672; NCT03785249), which bind the mutant residue and lock the oncoprotein in an inactive state; these agents have gained regulatory approvals in the corresponding mutant non-small-cell lung cancer setting and are under evaluation in multiple solid tumors (Clinical trial, NCT05067283). However, clinical responses are often incomplete and transient, with diverse resistance mechanisms—reactivation of GTPase signaling, pathway bypass, and emergence of secondary mutations—limiting the durability of benefit. Moreover, these agents address only a single mutant allele, leaving the majority of oncogenic GTPase variants across multiple isoforms and activating mutations without direct targeted options [474,475].

Beyond allele-specific covalent inhibition, broader approaches—including pan-isoform GTPase inhibitors, guanine nucleotide exchange factor inhibitors, and phosphatase-targeting agents—are in early-phase clinical or late preclinical development, frequently in combination regimens. While promising, these strategies still confront fundamental issues of feedback activation, narrow mutational coverage, and toxicity in GTPase-dependent normal tissues (clinical trial, NCT04111458), described in [476,477,478]. Signaling redundancy and adaptive feedback—the hallmarks that have frustrated decades of activity-based drug discovery against this GTPase family—operate entirely downstream of translation and are therefore invisible to translational control strategies. As with the disordered transcription factor oncogene discussed above, oncogenic GTPase expression is tightly coupled to specialized translational programs and ribosome features in cancer cells, suggesting that selectively dialing down translation of mutant GTPase mRNAs could complement or overcome the limits of activity-based inhibitors [477,479].

Viewed against this landscape, traditional therapeutic avenues for these two archetypal undruggable oncoprotein classes—small-molecule inhibitors of downstream pathways, transcriptional modulators, and protein degraders—have clearly established biological tractability and yielded some clinically promising agents, particularly the mutant-allele-specific covalent inhibitors and early oncoprotein-directed PROTAC degraders. Yet they remain constrained by target biology (structural disorder, adaptive signaling), limited mutational scope, resistance, and on-target toxicity [475]. RiboScreen™ offers a distinct and potentially synergistic strategy: by systematically profiling ribosome behavior on oncogene mRNAs and screening for compounds that selectively attenuate translation of hyperactive oncoproteins—whether a structurally disordered transcriptional driver or a panel of oncogenic GTPase variants spanning multiple activating alleles—it aims to control oncoprotein abundance upstream of protein function and degradation [4]. The translational control layer is indifferent to the structural disorder that defeats inhibitor design and insensitive to the downstream signaling rewiring that blunts activity-based agents; it operates on the mRNA itself, making it uniquely suited to targets whose resistance mechanisms emerge at the protein or pathway level. In principle, such mutation- and transcript-selective translational modulators could be deployed alone or in combination with allele-specific covalent inhibitors, PROTAC degraders, or epigenetic drugs, broadening the therapeutic arsenal for cancers driven by these disordered and signaling-redundant oncoproteins and extending targeted options to alleles and contexts not accessible to current activity-based agents [473].

In sum, clinical translation of modalities for diseases driven by PTCs has been only moderately successful, with very few approaches achieving durable benefit or regulatory approval in rare genetic disorders or in cancers carrying truncating mutations. This reflects the limited specificity of current readthrough drugs, sequence-context dependence, toxicity concerns (e.g., aminoglycosides), and the challenge of achieving sufficient restoration of full-length protein in affected tissues. Likewise, systemic modalities designed to normalize dysregulated protein expression have often fallen short of expectations, as seen for several targeted agents that modulate transcription or degradation but prove too narrow, pleiotropic, or poorly tolerated in complex disease settings. Against this backdrop, clinical development of RiboScreen technology has reached first-in-human status with artesunate in sJEB, and further development of artesunate derivatives with improved pharmacological properties is planned. While RiboScreen is currently being extended to additional dysregulated proteins, it is too early to draw firm conclusions on clinical translation for these programs.

Also, the RiboScreen^TM^ platform holds promise for developing small-molecule modalities in neurodegenerative and neurodevelopmental diseases, where we are currently nominating dysregulated protein candidates in Parkinson’s and Alzheimer’s disease. To the best of our knowledge, there are presently no clinical trials in which small molecules are explicitly designed to boost endogenous production of protective proteins (for example, components of the protein degradation machinery) or to selectively dampen synthesis of defined inflammatory mediators by acting at the level of translation in NDD; existing programs instead focus on aggregation, receptor signaling, or targeted protein degradation. These indications, therefore, represent an important test bed for first-in-class ribodrugs that fine-tune the translational output of disease-relevant proteins, guided by RiboScreen-enabled identification of ribosomal protein targets and ligands.

## 9. Conclusions

The development of novel drug discovery pipelines is most successful when it is firmly grounded in a well-understood biology of the molecular components underpinning the technology. In this manuscript, we have provided an integrated view of ribosome biology, emphasizing how ribosomal proteins can be re-conceptualized as regulatory nodes and druggable molecular valves that allow customized regulation of protein synthesis directly at the translation step. By aligning technological innovation with detailed mechanistic insight into ribosome composition, biogenesis, specialization, and ribosomal protein function, RiboScreen™ technology establishes a rational framework for protein therapy at the source—namely, the controlled modulation of protein output from disease-relevant mRNAs.

A central strength of the RiboScreen™ platform is its multi-layered precision-filtering pipeline, which progressively narrows candidates from in silico predictions to translationally meaningful leads. Beginning with the identification of a target ribosomal protein (TRP) and with structure-guided virtual docking and biophysical assessment, the workflow advances through yeast and human reporter systems, pathway-resolved proteomics, and high-content cell imaging, before finally interrogating activity in complex human tissue models and disease-relevant cell systems. At each step, filters are designed to enrich for compounds that bind defined ribosomal protein sites, selectively modulate a protein of interest, preserve global translation homeostasis, and exhibit favorable safety and pathway signatures, thereby ensuring both target specificity and clinical plausibility.

The translational impact of this ribosome-centric strategy is illustrated by two prototype programs. First, repurposing Artesunate via RiboScreen™ for severe junctional epidermolysis bullosa (sJEB) demonstrates first-in-human success in restoring full-length Lamb3 and promoting wound closure in an otherwise lethal genodermatosis. Second, the tropoelastin replenishment program identifies small molecules targeting rpL40e to enhance tropoelastin production, establishing a route toward systemic therapies for elastin-deficient conditions. Looking ahead, the same technological logic can be extended to PTC-driven rare diseases, oncogenic translation programs, and neurodegenerative disorders, positioning RiboScreen™ as a broadly applicable platform for precision control of protein synthesis across a spectrum of rare and prevalent diseases.

## Data Availability

No new data were created or analyzed in this study. Data sharing is not applicable to this article.

## Supplementary Materials

The following supporting information can be downloaded at https://www.mdpi.com/article/10.3390/biomedicines14071419/s1. Supplementary File: Challenges of applying maschine learning models to ribosomal proteins in virtual drug screening [480,481,482,483,484,485,486,487,488,489].
