# Supplementary material for: RiboScreenTM Technology Delivers Small-Molecule Ribodrugs to Convert Ribosomal Proteins into Molecular Valves for Tailored Protein Production Levels in Rare and Prevalent Disease
Source: Biomedicines. 2026 Jun 23;14(7):1419. doi: 10.3390/biomedicines14071419 (PMC13404128; doi:10.3390/biomedicines14071419)
Supplement: Supplementary file 1 [file biomedicines-14-01419-s001.zip › biomedicines-4307530-supplementary.pdf]

Challenges of applying maschine learning models to ribosomal proteins in virtual drug screening

Machine-learning approaches in molecular docking problems can have different critical caveats depending on their docking algorithms. A rather successful ansatz, which is implemented in AutoDock Vina[1,2] involves the construction of a scoring function assessing the quality of the docked pose based on pseudo free energies arising from statistical interatomic pair potentials, whose parameters are derived from large data sets of experimentally probed docked poses. On the downside, this approach does not result in physically relevant binding affinities and can only yield qualitative agreement with experiment. Graph theoretical approaches (e.g. EquiBind[3,4]) and generative diffusive search algorithms (e.g. DiffDock variants[5,6]) optimize docked poses purely on mathematical graph complementarity on the one hand and on local pharmacophore diffusion and subsequent potential energy minimization in the binding pocket on the other hand, attempting to resemble an induced fit mechanism[7] of the binding pocket and potential changes in binding pocket electrostatics due to apparent pKa shifts arising from the proximal ligand, as reported here[8]. Rather recently, a generative AI protocol has been reported to reliably build chemical compounds from scratch into pre-defined static binding pockets (IDOLpro[9]) with promising benchmark results. Many AI-based algorithms have been reported to struggle with distortions and over-compaction of the ligand compound artificially enforcing an "optimal", however chemically invalid pose (Fig. 4 in [10]). In the specific context of ribosomal proteins, all docking algorithms face an additional challenge: the non-trivial interplay in conformational dynamics between ribosomal subunits, RNA chains and protein constituents in the complex is expected to modulate local tertiary structure stability, dynamics and apparent pKa values in small ribosomal proteins as compared to available cryo-EM models of the ribosome, putting a strong emphasis on the conformational selection mechanism[7] and the transient availability of binding pockets for specific targets. Hence, machine-learning tools will likely guide researchers in the *in silico* pre-selection of possible pharmacophores for a given protein target at the ribosome surface based on an exhaustive conformational ensemble of the protein in its native environment, i.e. based on molecular dynamics simulations of the whole ribosome. Critical quality control of all proposed docked poses will always be imperative, even with prospective improvements of generative AI docking algorithms. A state-of-the-art evaluation of chemical affinities and apparent pKa shifts requires hybrid QM/MM approaches and constant pH molecular dynamics simulations of the docked pose, respectively.

References:

(1) Trott, O.; Olson, A. J. AutoDock Vina: Improving the Speed and Accuracy of Docking with a New Scoring Function, Efficient Optimization, and Multithreading. *J Comput Chem* **2010**, *31* (2), 455–461. https://doi.org/10.1002/jcc.21334.

(2) Buccheri, R.; Rescifina, A. High-Throughput, High-Quality: Benchmarking GNINA and AutoDock Vina for Precision Virtual Screening Workflow. *Molecules* **2025**, *30* (16), 3361. https://doi.org/10.3390/molecules30163361.

(3) Stärk, H.; Ganea, O.-E.; Pattanaik, L.; Barzilay, R.; Jaakkola, T. EquiBind: Geometric Deep Learning for Drug Binding Structure Prediction. **2022**. https://doi.org/10.48550/ARXIV.2202.05146.

(4) Li, Y.; Li, L.; Wang, S.; Tang, X. EQUIBIND: A Geometric Deep Learning-Based Protein-Ligand Binding Prediction Method. *DD&T* **2023**, *17* (5), 363–364. https://doi.org/10.5582/ddt.2023.01063.

(5) Corso, G.; Stärk, H.; Jing, B.; Barzilay, R.; Jaakkola, T. DiffDock: Diffusion Steps, Twists, and Turns for Molecular Docking. arXiv 2022. https://doi.org/10.48550/ARXIV.2210.01776.

(6) Herron, L.; Dakka, J.; Yao, K.; Shi, D.; Jerome, S. V. DiffDock-Glide: A Hybrid Physics-Based and Data-Driven Approach to Molecular Docking. *J. Chem. Inf. Model.* **2026**, *66* (10), 5641–5649. https://doi.org/10.1021/acs.jcim.5c01635.
(7) Vogt, A. D.; Di Cera, E. Conformational Selection or Induced Fit? A Critical Appraisal of the Kinetic Mechanism. *Biochemistry* **2012**, *51* (30), 5894–5902. https://doi.org/10.1021/bi3006913.
(8) Onufriev, A. V.; Alexov, E. Protonation and pK Changes in Protein–Ligand Binding. *Quart. Rev. Biophys.* **2013**, *46* (2), 181–209. https://doi.org/10.1017/S0033583513000024.
(9) Kadan, A.; Ryczko, K.; Lloyd, E.; Roitberg, A.; Yamazaki, T. Guided Multi-Objective Generative AI to Enhance Structure-Based Drug Design. *Chem. Sci.* **2025**, *16* (29), 13196–13210. https://doi.org/10.1039/D5SC01778E.
(10) Morehead, A.; Giri, N.; Liu, J.; Neupane, P.; Cheng, J. Assessing the Potential of Deep Learning for Protein–Ligand Docking. *Nat Mach Intell* **2025**, *8* (1), 32–41. https://doi.org/10.1038/s42256-025-01160-1.
